# Stage-matched conductive hydrogel with pH/ROS/MMP9 triple-responsive salidroside release for post-infarction myocardial repair

**DOI:** 10.1016/j.mtbio.2026.103016

**Published:** 2026-03-12

**Authors:** Jie Song, Zhongxiong Fan, Yakun Bo, Dilare Taiwaikuli, Jiayu He, Xing Zhang, Shaokai Ji, Yemin Chen, Huanhuan Ding, Heting Wu, Chao Wang, Baopeng Tang, Xianhui Zhou

**Affiliations:** a Department of Cardiac Pacing and Electrophysiology, The First Affiliated Hospital of Xinjiang Medical University, Urumqi, 830054, PR China; b Xinjiang Key Laboratory of Cardiac Electrophysiology and Remodeling, The First Affiliated Hospital of Xinjiang Medical University, Urumqi, 830054, PR China; c College of Pharmacy, Xinjiang Medical University, Urumqi, 830017, PR China; d School of Public Health, Xinjiang Medical University, Urumqi, 830017, PR China; e School of Pharmaceutical Sciences, Institute of Materia Medica, Xinjiang University, Urumqi, 830017, PR China

**Keywords:** Myocardial infarction, Smart hydrogel, Triple-responsive, Salidroside, Cardiac repair

## Abstract

Myocardial infarction (MI) triggers massive cardiomyocyte loss, inflammatory and fibrotic remodeling, and disruption of electrical conduction, which are difficult to reverse with a single therapeutic modality. Pathologically, the infarcted myocardium undergoes a sequential transition from local acidosis to oxidative/inflammatory injury and finally matrix metalloproteinase-driven scar formation. Guided by these stage-specific cues and the need to restore electrical continuity, we designed an injectable conductive hydrogel (PGO/CAM@Sal) that integrates a polypyrrole-based conductive network with pH/reactive oxygen species (ROS)/matrix metalloproteinase-9 (MMP9) triple-responsive salidroside delivery. The hydrogel matches myocardial stiffness, exhibits favorable injectability, self-healing and tissue adhesion, shows electrical conductivity comparable to native myocardium, and enables on-demand salidroside release under MI-mimicking conditions. In vitro, PGO/CAM@Sal effectively scavenges ROS, suppresses pro-inflammatory cytokine production, promotes M2 macrophage polarization and angiogenesis, and enhances connexin-43 expression and cardiomyocyte electrical coupling. In a rat MI model, local injection of PGO/CAM@Sal bridges the non-conductive infarct, stabilizes ventricular electrophysiology, improves conduction velocity and homogeneity, reduces arrhythmia vulnerability, and concurrently attenuates fibrosis, hypertrophy, and adverse left ventricular remodeling, leading to marked recovery of systolic function. Transcriptomic analysis suggests a potential involvement of the Mmp12/Cybb axis and related pathways involved in inflammation, oxidative stress, fibrosis, calcium handling, and ion transport. These molecular alterations were further validated by qRT-PCR and immunohistochemical analyses, collectively supporting the integrated therapeutic benefits observed. Accordingly, this stage-matched conductive smart hydrogel provides a comprehensive, spatiotemporally controlled strategy for precise post-MI repair.

## Graphical abstract

**Figure:**
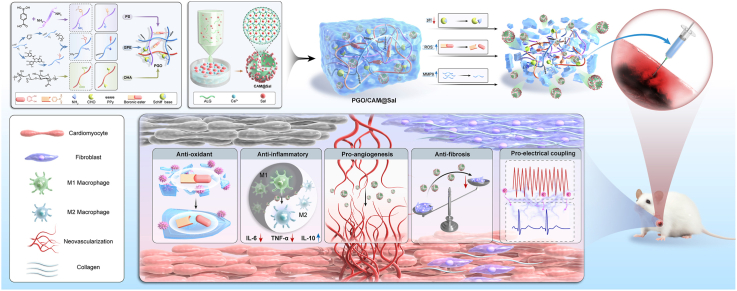

## 1 Introduction

Myocardial infarction (MI) remains a leading cause of mortality worldwide and continues to impose a substantial burden on global health [1]. MI is typically caused by acute coronary artery occlusion [2], which triggers oxidative stress, inflammatory cascades, and cardiomyocyte death. These pathological events lead to ventricular wall thinning, scar formation, adverse remodeling, and ultimately, heart failure [[3], [4], [5]]. Although current clinical strategies, including pharmacotherapy, percutaneous coronary intervention, and coronary artery bypass grafting, can partially alleviate symptoms and delay disease progression, they show limited efficacy in preventing adverse remodeling, restoring electromechanical coupling, and sustaining long-term cardiac function [[5], [6], [7]]. Heart transplantation remains the definitive treatment option for end-stage disease; however, its clinical application is limited by donor scarcity and immune rejection [8]. Accordingly, the development of innovative therapies capable of promoting myocardial repair and functional recovery is urgently required.

The pathological progression of MI follows a well-recognized three-stage pattern. In the initial phase, persistent cell death and lactate accumulation lower the local pH to approximately 6.5–6.8 [9]; The subsequent phase is characterized by inflammatory cell infiltration and excessive reactive oxygen species (ROS) production, further aggravating tissue damage [10]. In the later phase, M2 macrophages and myofibroblasts orchestrate anti-inflammatory and reparative responses, during which matrix metalloproteinase-9 (MMP9) upregulation gradually remodels the extracellular matrix (ECM) and drives scar formation [10,11]. Given this temporally evolving pathological landscape, therapeutic systems capable of providing mechanical support while actively responding to stage-specific microenvironmental cues are highly desirable for improving post-MI repair.

Injectable conductive hydrogels have emerged as a promising therapeutic platform for post-MI repair, as they not only reinforce the mechanically compromised ventricular wall but also reestablish electrical synchrony and impulse transmission across the infarct border zone [12,13]. A representative example is the polypyrrole (PPy)-grafted hydrogel reported by Zhang et al., which effectively restored electrical conduction in infarcted myocardium and mitigated adverse ventricular remodeling [12]. Building on this foundation, further integrated with bioactive agents has been shown to enhance therapeutic outcomes, enabling conductive hydrogels to simultaneously support structural reconstruction and functional reintegration [[14], [15], [16], [17]]. Nevertheless, conventional conductive hydrogels are generally incapable of adapting to the dynamic and heterogeneous pathological progression of MI, limiting the precision and timing of therapeutic interventions [3]. To address this challenge, stimulus-responsive hydrogels have been designed to release therapeutic bioactive agents in response to specific pathophysiological cues, such as changes in acidic pH, elevated ROS, or MMP activity [[18], [19], [20]]. Importantly, the pathological microenvironment of MI changes over time, progressing from initial acidic damage to oxidative and inflammatory stress, and finally to ECM remodeling mediated by MMP9. This dynamic evolution makes static delivery systems inadequate for targeted, stage-specific interventions.

Accordingly, delivering therapeutic agents that match the temporal biological demands of each pathological stage is essential. Salidroside (Sal), a bioactive glycoside extracted from *Rhodiola rosea* (molecular weight ≈ 300 Da) [21], is particularly s well suited for staged MI therapy. Its potent anti-inflammatory and antioxidant activities benefit the early and intermediate phases of MI, while its capacity to mitigate pathological fibrosis promotes more favorable tissue repair in the later phase [22,23]. However, its clinical translation is constrained by intrinsic drawbacks, including a short half-life, low bioavailability, and susceptibility to environmental degradation [24]. To overcome these limitations, we drew inspiration from the staged and adaptive tactics employed by Sun Wukong in *Journey to the West* during successive confrontations with the White Bone Demon—where each transformation was met with a phase-appropriate counterstrategy. Translating this concept into material design, the proposed hydrogel integrates Sal into a stage-matched pH/ROS/MMP9-responsive conductive network, enabling precisely timed and pathology-synchronized molecular interventions throughout MI progression. This strategy aims to maximize both structural remodeling and electrical-functional recovery of the injured myocardium.

**Scheme 1:**
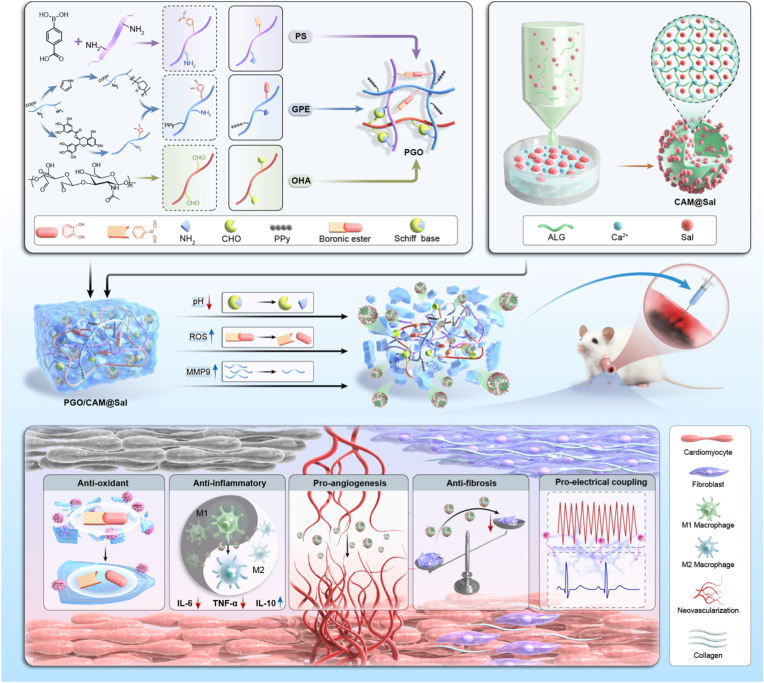
Schematic illustration of a pH/ROS/MMP9-responsive dynamic triple-network hydrogel for MI therapy.

To realize such stage-matched delivery, we constructed a multiresponsive injectable hydrogel by rationally aligning dynamic linkages with key pathological signals after MI. Silk fibroin (SF) is modified with 4-carboxyphenylboronic acid (CPBA) to obtain PS, while gelatin (G) is functionalized with epigallocatechin gallate (EGCG), followed by PPy polymerization through pyrrole–NH_2_ coupling to yield GPE. Subsequently, oxidized hyaluronic acid (OHA) is introduced. Its -CHO groups react with -NH_2_ groups in PS and GPE through Schiff base bonds (**pH-responsive**), CPBA and EGCG form boronate ester bonds (**ROS-responsive**), and the G backbone is susceptible to degradation by MMP9 (**MMP9-responsive**), resulting in a triple-responsive (pH/ROS/MMP9) hydrogel matrix termed PGO. Sal-loaded calcium alginate microspheres (CAM@Sal), prepared using a microfluidic technique and cross-linked with Ca^2+^, are incorporated into the PGO hydrogel matrix to construct the composite hydrogel PGO/CAM@Sal. In the present study, we systematically characterize the physicochemical, multistimuli-responsive and electrical properties of PGO/CAM@Sal, and evaluate its ability to orchestrate inflammatory, oxidative, angiogenic, fibrotic and electrophysiological remodeling in vitro and in a rat MI model (Scheme 1). Furthermore, bulk RNA sequencing–based transcriptomic profiling is employed to delineate the myocardial signaling pathways engaged by PGO/CAM@Sal and to relate these programs to its integrated structural and electrical benefits.

## 2 Results and discussion

### 2.1 Design and fabrication of PGO/CAM@Sal hydrogel

**Fig. 1:**
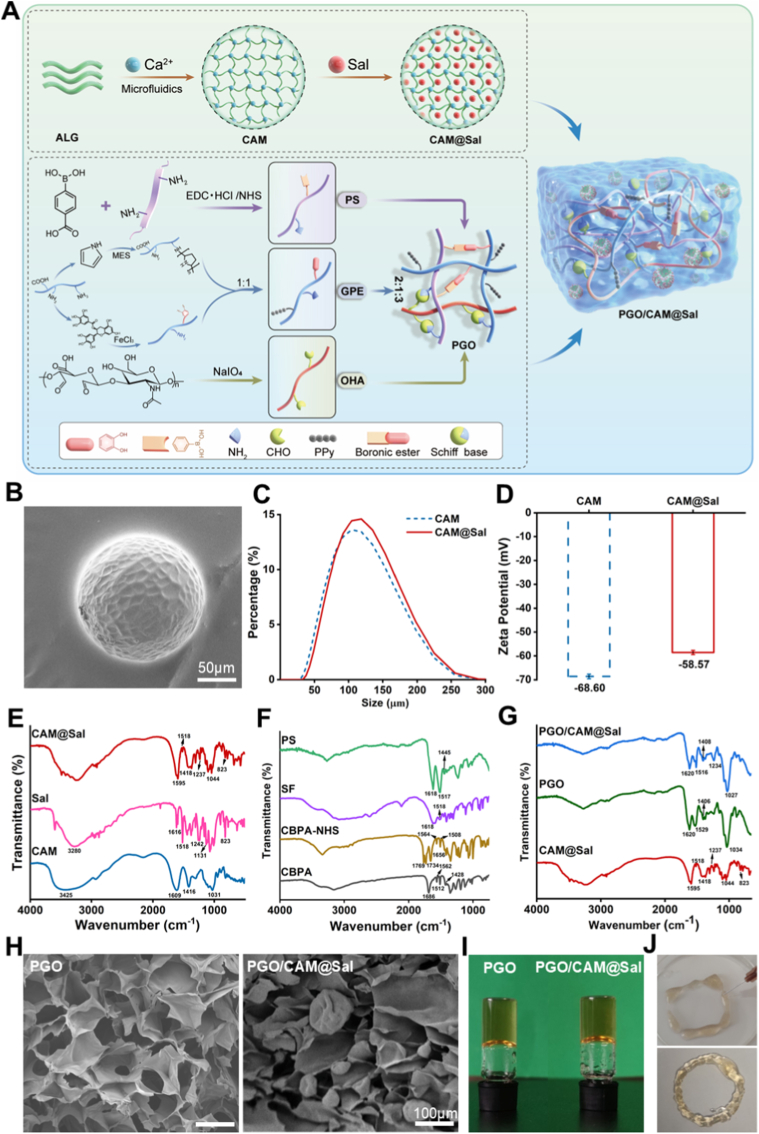
**Design and characterization of PGO/CAM@Sal hydrogels.** A) Schematic illustration of PGO/CAM@Sal fabrication. B) SEM images of CAM@Sal. C) Particle size distribution of CAM and CAM@Sal. D) Zeta potential of CAM and CAM@Sal. E) FTIR of CAM, Sal and CAM@Sal. F) FTIR of CPBA-NHS, SF and CPBA-SF. G) FTIR of PGO and PGO/CAM@Sal. H) SEM images of different hydrogels. I) Photos of PGO and PGO/CAM@Sal hydrogels. J) Photos of injectable properties of PGO/CAM@Sal hydrogel.

CAM was prepared using microfluidic technology and subsequently loaded with Sal via immersion to form Sal-loaded microspheres (CAM@Sal) (Fig. 1A). A cell counting kit-8 (CCK-8) assay demonstrated that a 1 mg/mL concentration of Sal optimally maintains biocompatibility (Fig. S1A). Bright-field microscopy revealed that CAM appeared as transparent spherical particles (Fig. S2A), and confocal laser scanning microscopy confirmed uniform internal loading of Rhodamine B, suggesting that Sal was evenly distributed (Fig. S2B). Scanning electron microscopy (SEM) images showed that blank microspheres exhibited smooth and dense surfaces (Fig. S2C), whereas slight surface wrinkling appeared after Sal loading (Fig. 1B, Fig. S2D). Notably, low-magnification SEM images of blank CAM were not included, which may partially constrain direct morphological comparison at the population level. Nonetheless, particle size analysis demonstrated an increase in the average diameter from 104.58 μm for CAM to 118.82 μm for CAM@Sal (Fig. 1C), confirming successful drug encapsulation. In parallel, the zeta potential shifted from −68.60 mV to −58.57 mV (Fig. 1D), suggesting partial neutralization of surface charges by Sal. The encapsulation efficiency (EE) was measured to be 72.76% (Fig. S1B).

To further assess morphological stability of microspheres, the hydrodynamic diameter and zeta potential of CAM and CAM@Sal were monitored at 0, 6, 12 and 24 h in PBS. No significant changes were detected over time (Table S1), indicating that both formulations maintained good colloidal and structural stability in a hydrated environment. Importantly, particle size distribution analysis demonstrated acceptable overall uniformity, and the spherical integrity of blank CAM was consistently confirmed by bright-field and confocal microscopy. Collectively, these results indicate that CAM@Sal microbeads possess sufficient structural stability and uniformity to support subsequent hydrogel incorporation and biological evaluation.

Systematic comparison of PGO hydrogels with PS: GPE: OHA ratios ranging from 2:1:1 to 2:1:4 demonstrated that the 2:1:3 formulation achieved the optimal balance among gelation time, electrical conductivity, and compressive modulus, supporting its selection as the optimal formulation for subsequent in vivo studies (Table S2). Fourier transform infrared spectroscopy (FTIR) confirmed the successful fabrication of the material and its composite structure (Fig. 1E–G). Characteristic Sal peaks (1518, 1242, and 823 cm^−1^) were observed in CAM@Sal, and the CAM peak at 1609 cm^−1^ shifted slightly to 1595 cm^−1^, indicating that Sal was embedded and stabilized through hydrogen bonding and electrostatic interactions (Fig. 1E). PS displayed amide and aromatic ring absorptions at 1618, 1517, and 1445 cm^−1^ (Fig. 1F), while GP exhibited a C–C vibration peak at 1451 cm^−1^ accompanied by enhanced amide signals (Fig. S3A). GE showed composite peaks at 1629 and 1541 cm^−1^ (Fig. S3A), and OHA displayed an aldehyde C=O peak at 1720 cm^−1^ (Fig. S3B), collectively confirming successful assembly. PGO/CAM@Sal spectra retained the characteristic peaks of PS, GP, GE, and OHA at 1620, 1529, 1406, and 1034 cm^−1^, respectively, with additional CAM@Sal peaks at 1516 and 1234 cm^−1^ (Fig. 1G), demonstrating successful formation of the composite hydrogel.

SEM images revealed a porous, cross-linked three-dimensional (3D) network structure with embedded microspheres (Fig. 1H). The slightly irregular appearance of CAM@Sal observed after embedding is most likely associated with SEM sample preparation procedures, including freezing and dehydration, which may induce partial collapse of hydrogel-based microbeads due to water desorption under confined conditions [25]. Inverted-vial tests showed that PGO/CAM@Sal gelled in approximately 20 s, which was faster than PGO alone (30 s), thereby enabling rapid in situ formation (Fig. 1I). Additionally, the hydrogel was smoothly extruded through a 27-G needle, demonstrating excellent injectability (Fig. 1J).

### 2.2 Multifunctional characteristics of PGO/CAM@Sal hydrogel

**Fig. 2:**
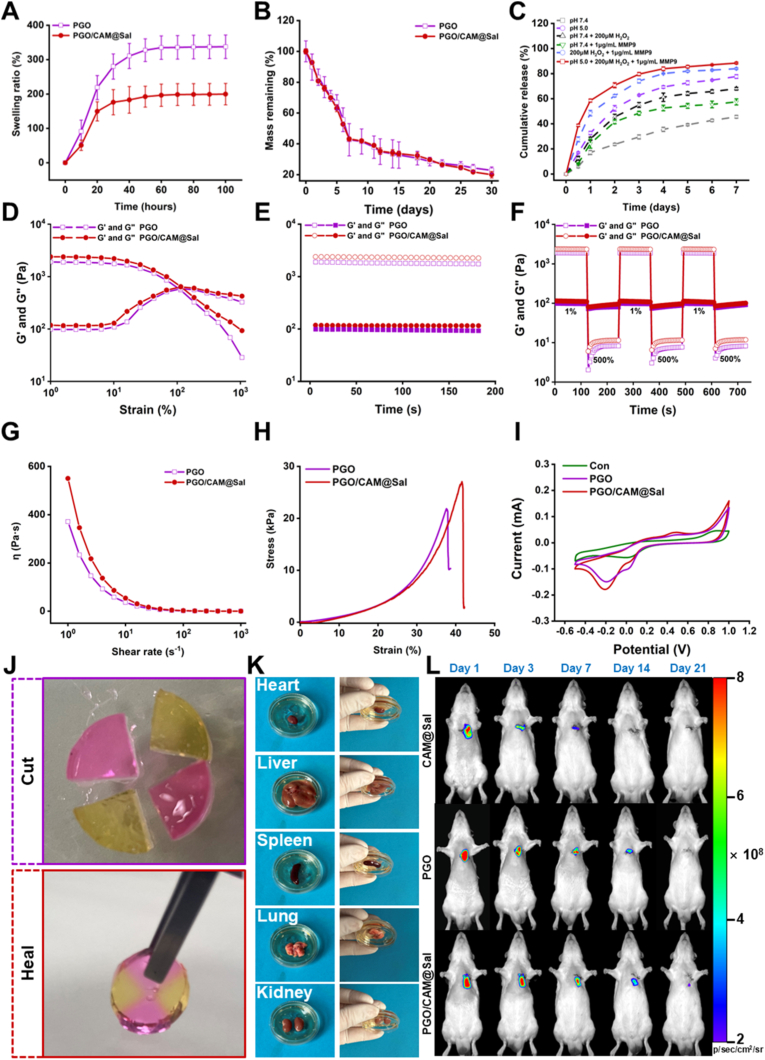
**Properties of PGO/CAM@Sal Hydrogels.** A) Swelling ratio of hydrogels in PBS (n = 3). B) Mass remaining of different hydrogels in PBS and Collagenase Solution (n = 3). C) Release profiles of Sal from PGO/CAM@Sal hydrogel under different conditions (n = 3). D) G′ and G″ of the hydrogels under different oscillatory strains. E) G′ and G″ of two hydrogels under dynamic time sweep (1% strain, 1 Hz). F) Self-healing properties of hydrogels under alternating strain. G) Viscosity of hydrogels as a function of shear rate. H) Stress-strain curves of different hydrogels. I) CV curves of different hydrogels. J) Self-Healing process of hydrogels loaded with fluorescent dyes. K) Adhesion performance of hydrogels on vital organs. L) IVIS images of Cy5.5-labeled hydrogels at days 1, 3, 7, 14, and 21 post-injection.

PGO/CAM@Sal hydrogel provided a spatiotemporally controlled Sal release through multi-responsive degradation, with the pore structure directly affecting drug diffusion. Swelling studies showed that both hydrogels reached equilibrium within 70 h, with PGO/CAM@Sal exhibiting a slightly higher swelling ratio (335.73%) than PGO (198.67%) (Fig. 2A), likely because the incorporation of CAM increased the overall hydrophilicity of the hydrogel network. Degradation in PBS containing 0.2 U/mL collagenase (to simulate post-MI enzymatic degradation) resulted in >50% mass loss within 7 days; after 30 days, residual masses were 22.75% for PGO and 20.01% for PGO/CAM@Sal (Fig. 2B), supporting rapid acute-phase Sal release while enabling continued delivery for myocardial repair. SEM revealed structural collapse and reduced network order in the degraded hydrogels (Fig. S4). Drug release experiments demonstrated triple responsiveness of PGO/CAM@Sal to pH, ROS, and MMP9 stimuli (Fig. S5). Cumulative release over 7 days reached 77.72%, 68.01%, and 57.49% under pH 5.0, 200 μM H_2_O_2_, and 1 μg/mL MMP9, respectively, with a maximum of 88.43% under combined stimuli (Fig. 2C), confirming stage-specific delivery. Interestingly, compared with CAM@Sal microbeads alone, which exhibited a rapid early burst release and reached near-complete salidroside release within 7 days (Fig. S6), PGO/CAM@Sal displayed a markedly delayed release kinetics, with limited cumulative release unless exposed to pathological triple stimuli. This difference indicates that the PGO hydrogel matrix effectively suppresses premature drug diffusion and enables a more sustained and controlled release profile. Such a release behavior is better aligned with the temporal evolution of oxidative stress and inflammation after myocardial injury, thereby favoring prolonged therapeutic efficacy rather than rapid drug depletion.

Rheological analysis indicated that the hydrogels maintained a stable network under low strain but collapsed when strain exceeded the critical point (G′ < G″) due to cleavage of Schiff-base and boronate-ester bonds (Fig. 2D). Incorporation of microspheres did not significantly alter the gel–sol transition. Time-sweep measurements (1% strain, 1 Hz) showed that G′ > G″ remained stable over time (Fig. 2E), indicating rapid network formation and sustained structural integrity. Alternating-strain tests demonstrated self-healing after high-strain (500%) damage (Fig. 2F). The shear-thinning behavior ensured injectability and enabled in situ structural reconstruction (Fig. 2G). Compression tests (Fig. 2H) revealed that PGO/CAM@Sal exhibited a slightly higher compressive modulus than PGO, reaching 27.93 kPa (Fig. S7A), which fell within the physiological range of the adult myocardium (8–60 kPa) [26] and was sufficient to support cardiac contraction. While the mechanical properties of individual CAM and CAM@Sal microparticles were not independently quantified, the preserved and slightly enhanced bulk mechanical performance of the composite hydrogel after microbead incorporation indirectly suggests that the embedded microparticles possess adequate mechanical integrity and compatibility with the hydrogel matrix.

Hydrogel conductivity was assessed using a four-point probe and electrochemical analysis. Non-PPy PGO showed a conductivity of 3.48 × 10^−5^ S/cm, whereas PPy-containing PGO and PGO/CAM@Sal reached 7.35 × 10^−4^ and 7.44 × 10^−4^ S/cm (Fig. S7B), respectively, values comparable to those of native myocardium (≈10^−4^ S/cm) [27]. Consistent with the conductivity results, cyclic voltammetry also showed that PPy-containing hydrogels exhibited higher capacitance (Fig. 2I), confirming their enhanced electroactive performance. Self-healing and adhesion tests using FITC-labeled (yellow) and Rhodamine B-labeled (red) PGO/CAM@Sal hydrogels revealed complete interface fusion within 3 h, demonstrating excellent self-healing ability (Fig. 2J). Tissue adhesion experiments confirmed stable attachment to centrifuge tubes (Fig. S8) and mouse organs (Fig. 2K), ensuring sustained drug delivery to infarcted regions. Meanwhile, to evaluate the in vivo degradability and drug retention behavior of the hydrogels, in vivo imaging system (IVIS) was performed for CAM@Sal, PGO, and PGO/CAM@Sal at days 1, 3, 7, 14, and 21 post-injection (Fig. 2L). The CAM@Sal group exhibited a rapid decline in fluorescence intensity, with signals nearly completely quenched by day 14, indicating fast in vivo clearance. In contrast, the PGO/CAM@Sal hydrogel showed persistent fluorescence signals up to day 21 post-injection (Fig. S9), reflecting slow degradation and sustained drug retention in vivo. Importantly, the in vivo fluorescence decay profile was consistent with the extended in vitro degradation results (beyond 28 days), supporting the long-term degradability and sustained drug-retention capability of the PGO/CAM@Sal hydrogel.

Collectively, these results demonstrate that the PGO/CAM@Sal hydrogel integrates favorable injectability, rapid in situ gelation, multi-responsive drug release, controlled degradation, stable conductivity, and mechanical properties within the physiological range of the adult myocardium, supporting its potential as a multifunctional injectable platform for myocardial repair.

### 2.3 Biocompatibility evaluation of PGO/CAM@Sal hydrogel

**Fig. 3:**
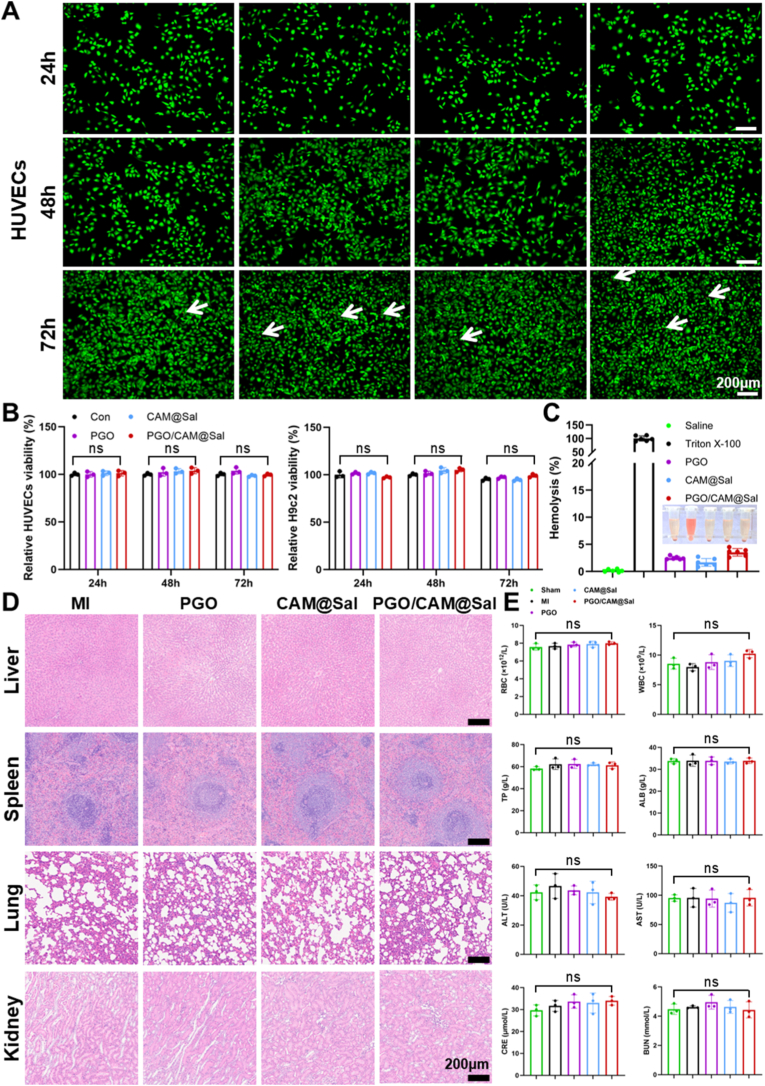
**In vitro and in vivo biocompatibility evaluation of the PGO/CAM@Sal hydrogel.** A) Live/dead staining of HUVECs after incubation with hydrogel extracts (green: live cells; red: dead cells). B) Cell viability of HUVECs and H9c2 determined by CCK-8 assay after exposure to hydrogel extracts; untreated cells served as controls (n = 3). C) Quantitative analysis of hemolysis ratio of different hydrogels. D) H&E staining of major organs (liver, spleen, lung, and kidney) harvested from rats in different treatment groups. E) Complete blood count and serum biochemical parameters after 28 days of treatment (n = 3). (For interpretation of the references to color in this figure legend, the reader is referred to the Web version of this article.)

Live/dead staining revealed high cell viability and preserved morphology of both human umbilical vein endothelial cells (HUVECs, Fig. 3A) and rat embryonic cardiomyocytes (H9c2, Fig. S10) cultured on PGO/CAM@Sal hydrogels. CCK-8 assays showed no significant cytotoxicity over 24–72 h (Fig. 3B). Hemolysis testing demonstrated <5% hemolysis for all hydrogel formulations (Fig. 3C), satisfying clinical safety standards. In vivo biocompatibility assessment via histology and blood biochemistry revealed no evident inflammation or tissue damage 28 days after hydrogel injection (Fig. 3D), and blood indices remained within normal physiological ranges (Fig. 3E), confirming favorable biosafety.

### 2.4 Anti-inflammatory and anti-oxidant effects of PGO/CAM@Sal hydrogel in vitro

**Fig. 4:**
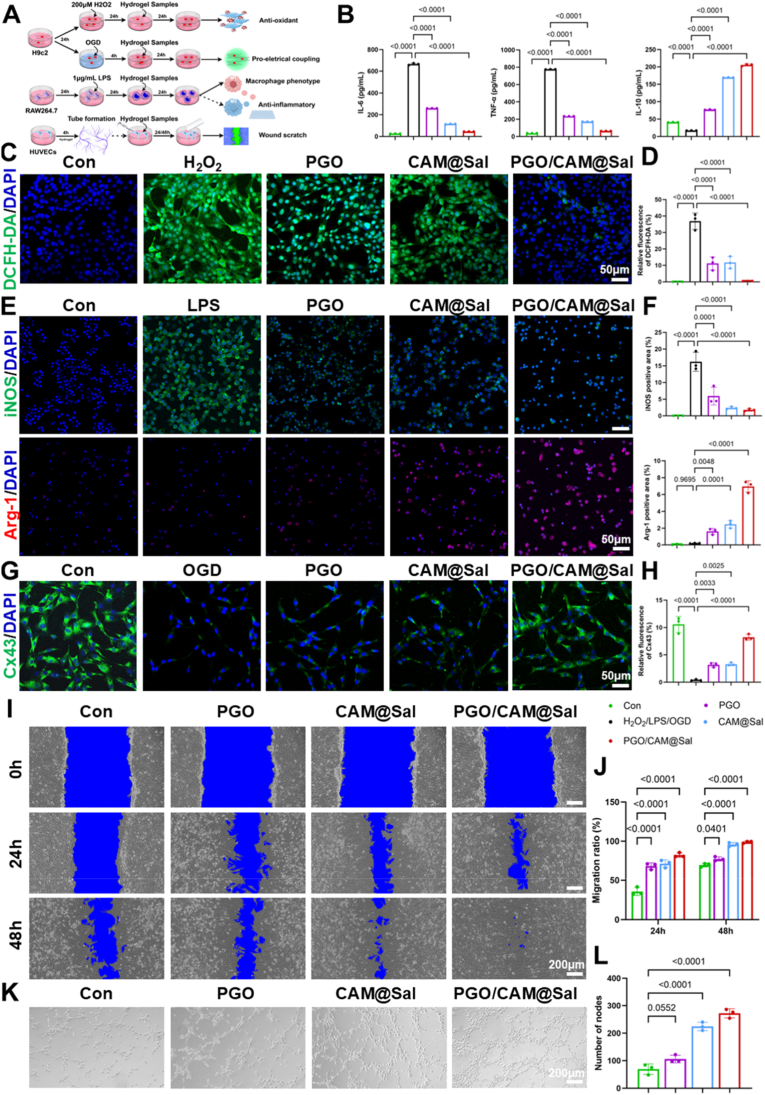
**Effects of PGO/CAM@Sal hydrogels on anti-inflammatory, antioxidant, electrophysiological and pro-angiogenic activities in vitro.** A) Schematic illustration of in vitro functional validation. B) Quantification of pro-inflammatory (IL-6, TNF-α) and anti-inflammatory (IL-10) cytokines in RAW264.7 cells by ELISA. C) DCFH-DA (green) staining of H9c2 cells treated with H_2_O_2_ (200 μM) and different hydrogel extracts. D) Quantitative analysis of ROS elimination in vitro (n = 3). E) Immunofluorescence staining of iNOS (green) and Arg-1 (red) in RAW264.7 cells treated with LPS (1 μg/ml) and different hydrogel extracts. F) Quantitative analysis of macrophage polarization in vitro (n = 3). G) Immunofluorescence staining of Cx43 (green) in H9c2 cells subjected to OGD and treated with different hydrogel extracts. H) Quantitative analysis of Cx43 expression (n = 3). I) Representative images of HUVECs treated with different hydrogel extracts at 0, 24, and 48 h. J) Quantitative analysis of migration ratio at each time point (n = 3). K) Representative Images of HUVECs forming tubes in vitro. L) Quantification of tube nodes (n = 3). (For interpretation of the references to color in this figure legend, the reader is referred to the Web version of this article.)

During the inflammatory phase of MI, the recruitment of inflammatory cells and neutrophils accelerated ECM degradation and proinflammatory cytokine release, thereby impeding myocardial repair [3]. IL-6 and TNF-α were prototypical proinflammatory cytokines, whereas IL-10 served as a major anti-inflammatory mediator [28]. To evaluate the anti-inflammatory properties of hydrogels, RAW264.7 mouse macrophages were stimulated with 1 μg/mL LPS (Fig. 4A). LPS markedly increased IL-6 (665.53 pg/mL) and TNF-α (774.40 pg/mL), whereas hydrogel treatment substantially reduced these cytokines, with PGO/CAM@Sal achieving the most pronounced suppression (IL-6:44.49 pg/mL; TNF-α: 62.49 pg/mL) (Fig. 4B). IL-10 secretion increased most prominently in the PGO/CAM@Sal group (205.42 pg/mL) (Fig. 4B), indicating enhanced modulation of inflammatory response through coordinated regulation of pro and anti-inflammatory cytokines.

Notably, both PGO and PGO/CAM@Sal contain intrinsic anti-inflammatory components, including EGCG, phenylboronic acid-modified gelatin and hyaluronic acid, which have been reported to modulate inflammatory signaling and macrophage activation [14,[29], [30], [31]]. However, the substantially stronger cytokine regulation observed in the PGO/CAM@Sal group suggests that the bioactivity cannot be attributed to a single component. Instead, it reflects a synergistic mechanism in which sustained Sal release amplifies and prolongs the baseline anti-inflammatory effects provided by the conductive hydrogel matrix.

Excessive ROS generation is a central pathological mechanism underlying MI and contributes to cardiomyocyte injury [32]. An oxidative stress model was induced in H9c2 cardiomyocytes using H_2_O_2_ (200 μM) (Fig. 4A), and ROS-scavenging capacity was quantified using 2′,7′-Dichlorodihydrofluorescein diacetate (DCFH-DA) staining (Fig. 4C). Hydrogel treatment significantly reduced intracellular ROS levels. Both PGO (11.16%) and CAM@Sal (11.72%) exhibited moderate antioxidant activity, whereas PGO/CAM@Sal markedly reduced ROS levels to 0.45% (Fig. 4D), demonstrating synergistic ROS-scavenging performance arising from boronate-ester bonds in PGO and Sal in CAM@Sal.

The transition of macrophages from pro-inflammatory M1 to anti-inflammatory M2 phenotypes is essential for tissue repair [33]. Immunofluorescence staining demonstrated that LPS treatment elevated inducible nitric oxide synthase (iNOS, M1 marker) expression to 16.21% and reduced arginase-1 (Arg-1, M2 marker) expression to 0.19% (Fig. 4E and F). Hydrogel treatment downregulated iNOS and upregulated Arg-1 to varying degrees, with PGO/CAM@Sal producing the strongest effect (iNOS: 1.78%; Arg-1:6.96%) (Fig. 4F). Although PGO itself exhibits ROS-responsive scavenging capability through reversible boronate ester chemistry [14,[29], [30], [31]], the near-complete suppression of intracellular ROS in the PGO/CAM@Sal group indicates an additive and potentially synergistic antioxidant mechanism. At the molecular and cellular levels, the conductive hydrogel matrix offers rapid ROS buffering and baseline immunomodulation, whereas sustained Sal release further reinforces these effects by attenuating intracellular oxidative stress and inflammatory signaling. Sal may exert prolonged antioxidant protection by modulating mitochondrial redox homeostasis and suppressing NADPH oxidase–associated ROS generation, among other pathways, thereby providing sustained relief from oxidative injury [34,35].

### 2.5 Electrophysiological and pro-angiogenic effects of PGO/CAM@Sal hydrogel in vitro

PPy imparts excellent conductivity, enabling conductive injectable hydrogels to establish an electrical bridge across infarct regions, thereby restoring cardiac electrical synchrony [36]. Connexin-43 (Cx43) is a key gap junction protein that facilitates action potential propagation and synchronizes cardiomyocyte contraction [17]. In vitro, immunofluorescence staining for Cx43 (Fig. 4G) revealed significantly enhanced expression at the plasma membranes and cell–cell junctions in the PGO/CAM@Sal group (8.23%) compared with the OGD group (0.41%) (Fig. 4H). While improved Cx43 expression is directly associated with the conductive properties of PPy-containing hydrogels, it should be noted that oxidative stress and inflammatory cytokines are well-established inhibitors of gap junction integrity and electrophysiological stability [37]. Therefore, the pronounced restoration of Cx43 expression in vitro is likely the result of coordinated electrical bridging by the PPy network and microenvironmental normalization mediated by Sal.

Effective myocardial repair relies on robust angiogenesis, with endothelial cell–mediated vascularization playing a central role [38]. The pro-angiogenic effects of the hydrogels were assessed using scratch and tube formation assays on HUVECs (Fig. 4A). In the scratch assays, the hydrogel-treated groups exhibited markedly enhanced migration at 24 h, and the PGO/CAM@Sal group achieved complete closure after 48 h (Fig. 4I). Quantitative analysis confirmed these observations (Fig. 4J), and the superior migration observed in the CAM@Sal group was attributed to Sal-promoted endothelial cell proliferation and motility [39]. Tube formation assays (Fig. 4K) revealed a notable rise in nodes (Fig. 4L), junctions, and branches (Fig. S11) within the PGO/CAM@Sal group, highlighting its pro-angiogenic potential due to Sal-induced angiogenesis [21,40].

Taken together, these results indicate that the overall therapeutic benefits observed in the PGO/CAM@Sal system arise from a coordinated interplay between electrical conduction restoration and pharmacological microenvironment modulation. Electrical integration primarily contributes to improved cardiomyocyte coupling and signal propagation, whereas Sal-mediated antioxidant and anti-inflammatory effects alleviate pathological barriers that otherwise impair electrical and vascular remodeling. Rather than acting independently, these mechanisms operate synergistically to promote comprehensive myocardial repair.

### 2.6 Anti-remodeling and anti-fibrotic effects of PGO/CAM@Sal hydrogel in vivo

**Fig. 5:**
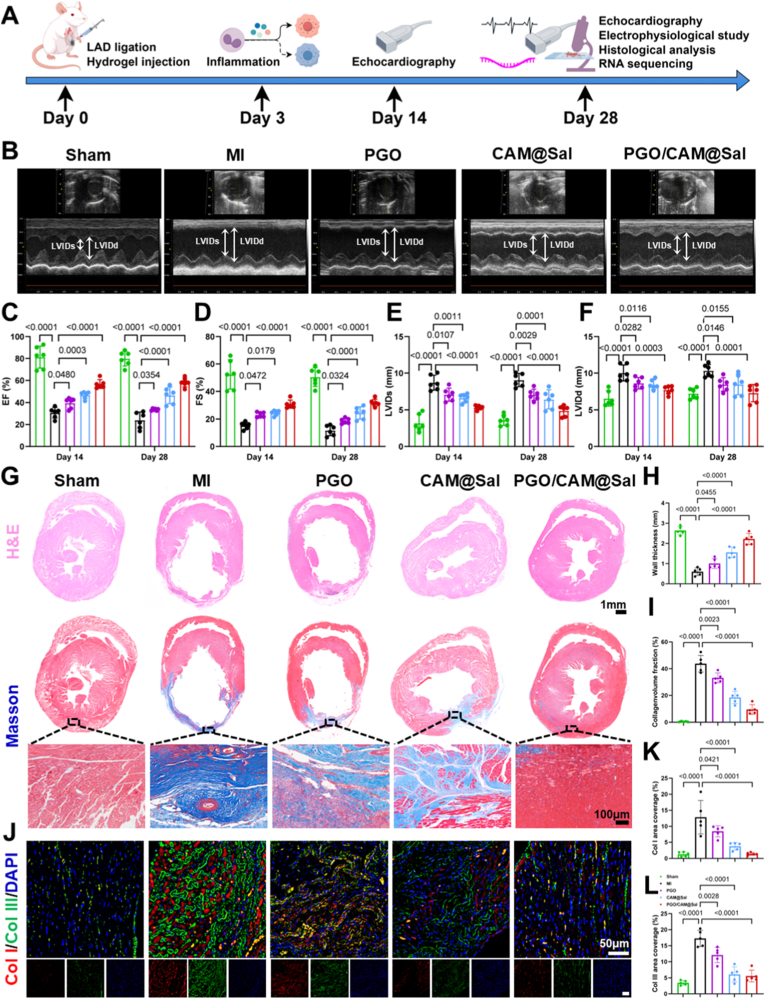
**PGO/CAM@Sal Hydrogels improve cardiac function and mitigate myocardial fibrosis.** A) Schematics of in vivo experiments. B) Representative echocardiograms of each group at 28 days post injection. C ∼ F) Quantitative echocardiographic assessment of key cardiac function indicators at 14 and 28 days post hydrogel treatments (n = 6), including EF (C), FS (D), LVIDs (E), LVIDd (F). G) H&E and masson trichrome staining of infarcted myocardium at 28 days after different treatments. H ∼ I) Quantitative assessment of infarct wall thickness (H) and collagen volume fraction (I) at 28 days post hydrogel treatments (n = 5). J ∼ L) Immunofluorescence staining of infarcted myocardium (J) and quantitative analysis of collagen I (K) and collagen III (L) (n = 5).

Building on the favorable physicochemical properties, biocompatibility, and in vitro good biological functions of the hydrogels, their therapeutic potential was evaluated for mitigating pathological left ventricular remodeling in a rat MI model. The experimental design and timeline are shown in Fig. 5A. MI was induced by ligating the left anterior descending coronary artery, and successful model establishment was confirmed by significant ST-segment elevation on electrocardiogram (ECG, Fig. S12). The rats were randomly assigned to the Sham, MI, PGO hydrogel, CAM@Sal hydrogel, and PGO/CAM@Sal hydrogel treatment groups. The hydrogels were injected into multiple infarct sites using a 30-G needle (Fig. S13).

Echocardiography was performed at two and four weeks after treatment. Echocardiographic analysis revealed pronounced differences in cardiac function between the groups (Fig. 5B). After two weeks, the MI group showed marked cardiac dysfunction and left ventricular remodeling, evidenced by a significantly reduced ejection fraction (EF, 30.70%) and fractional shortening (FS, 15.26%), along with increased left ventricular internal diameter at end-systole (LVIDs, 8.70 mm) and end-diastole (LVIDd, 10.03 mm) (Fig. 5C–F). The PGO/CAM@Sal hydrogel-treated rats exhibited significant improvements in EF (56.72%) and FS (30.88%), along with reductions in LVIDs (5.32 mm) and LVIDd (7.71 mm), due to the mechanical support of the hydrogel (Fig. 5C–F). At four weeks, the MI rats exhibited further functional decline, whereas all hydrogel-treated groups demonstrated improved cardiac performance, indicating sustained therapeutic effects.

Histological analysis after 28 days confirmed the long-term therapeutic benefits. Hematoxylin and eosin (H&E) staining revealed ventricular enlargement and wall thinning in the MI group, whereas all hydrogel-treated hearts exhibited increased wall thickness (Fig. 5G), with the greatest improvement observed in the PGO/CAM@Sal group (2.22 mm) (Fig. 5H). This structural preservation was attributable to the boronate-ester bonds with the hydrogel and sustained Sal release, which synergistically scavenged ROS in the infarct regions, thereby improving the local microenvironment and effectively limiting infarct expansion.

Myocardial fibrosis is a critical component of left ventricular remodeling. Masson's trichrome staining indicated reduced fibrosis in the PGO (33.13%) and CAM@Sal (18.54%) groups compared with the MI group (43.80%) (Fig. 5G–I), attributable to the anti-fibrotic effects of gallic acid [41] and Sal [42]. Notably, the PGO/CAM@Sal group showed the greatest reduction in fibrosis (9.52%), consistent with the staged Sal release and gradual hydrogel degradation. Excessive collagen deposition contributed to fibrosis, with collagen I promoting matrix accumulation, and collagen III maintaining tissue elasticity [43]. Although increased levels of collagen III partially alleviated fibrosis, excessive deposition could cause hypertrophy, dilatation, and heart failure [44]. Immunofluorescence staining for collagen I and collagen III (Fig. 5J) revealed a substantial reduction in the PGO/CAM@Sal group (collagen I: 1.40%, collagen III: 5.57%) compared with the MI group (collagen I: 12.83%, collagen III: 17.28%) (Fig. 5K and L), confirming the effective anti-fibrotic action.

The heart-weight-to-tibia-length ratio (HW/TL) was measured to evaluate adverse remodeling. PGO/CAM@Sal-treated rats exhibited significantly lower HW/TL values (31.41 mg/mm) than the MI group (41.35 mg/mm) (Fig. S14A and B). Wheat germ agglutinin (WGA) staining of peri-infarct cardiomyocytes demonstrated that hydrogel treatment, particularly PGO/CAM@Sal (201.00 μm^2^), significantly suppressed hypertrophy compared to MI (508.58 μm^2^) (Fig. S15A and B). Collectively, PGO/CAM@Sal effectively attenuated adverse remodeling and promoted structural recovery of the infarcted myocardium.

### 2.7 Protective effect of PGO/CAM@Sal hydrogel on cardiac electrophysiological stability in vivo

MI-induced fibrosis disrupts electrical connectivity among viable cardiomyocytes, promoting desynchronization and arrhythmias [45]. To address this pathological discontinuity, conductive biomaterials have been shown to bridge the electrical gap between healthy and infarcted myocardium, restore continuity, and reduce arrhythmic events [17,46]. In this context, given the excellent in vitro conductivity of the PGO/CAM@Sal hydrogel, its impact on cardiac electrophysiology 28 days post-MI was evaluated.

**Fig. 6:**
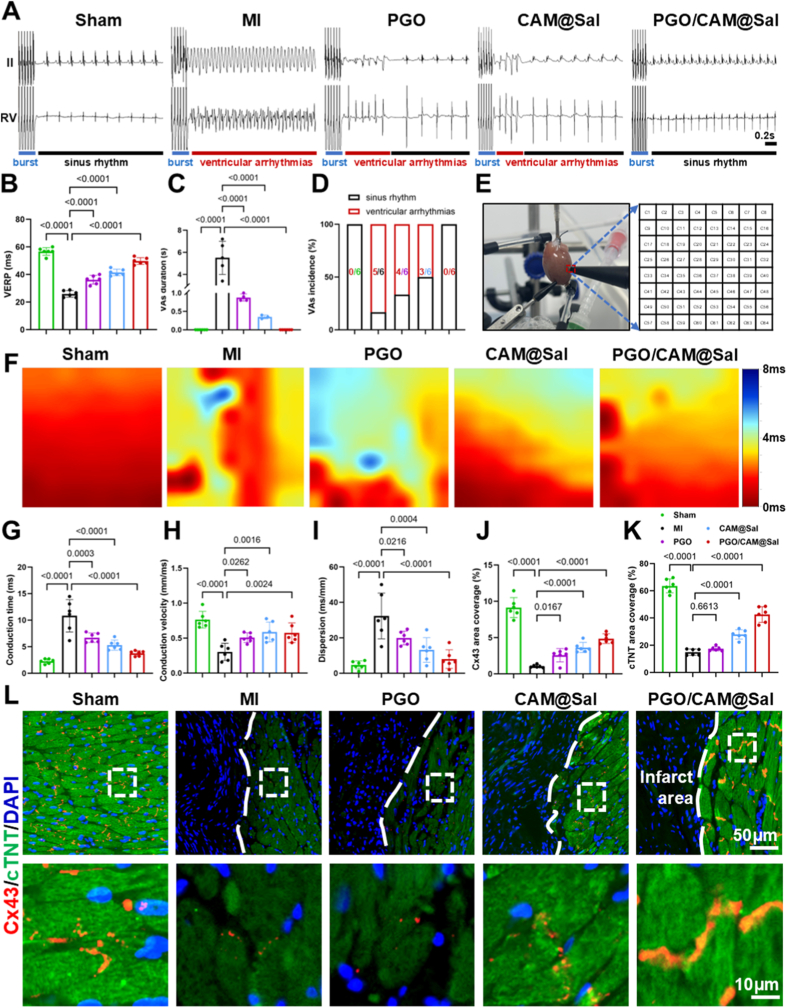
**PGO/CAM@Sal Hydrogels reduces ventricular arrhythmia susceptibility and improves myocardial conduction in vivo.** A) Typical images of VAs induced by burst pacing at 50 ms S_1_-S_1_ interval among different hydrogels. B ∼ D) VAs susceptibility-related indicators including VERP (B), VAs duration (C) and VAs incidence (D) (n = 6). E) Schematic illustration of electrical conduction measurement on the Langendorff-perfused heart using a 64-channel electrode array (8 × 8 grid, 0.55 mm spacing; epicardial stimulation: 2 mA; 5 Hz at the left ventricular apex; electrode positioned at the infarct–normal myocardial border zone). F) Representative epicardial activation maps across groups, recorded from healthy to infarcted regions (earliest activation is shown in red, latest in blue, with scale bar values indicating activation time). G ∼ I) Quantitative analysis of conduction time (G), conduction velocity (H), and conduction dispersion (I) at 28 days post-treatment. J ∼ L) Quantitative analysis of Cx43 (J) and cTnT (K) expression in infarcted regions (n = 6), and representative immunofluorescence images (L) of Cx43 (red) and cTNT (green) with DAPI-stained nucleus (blue) at 28 days post-MI. (For interpretation of the references to color in this figure legend, the reader is referred to the Web version of this article.)

In vivo electrophysiological assessments revealed that PGO and CAM@Sal hydrogels moderately shortened the duration of ventricular arrhythmias (VAs), whereas PGO/CAM@Sal nearly abolished arrhythmias (Fig. 6A). All hydrogel-treated groups reduced ventricular effective refractory period (VERP) (Fig. 6B), VAs duration (Fig. 6C), and VAs incidence (Fig. 6D), with PGO/CAM@Sal demonstrating the strongest effect. From a pathophysiological perspective, VERP shortening is a characteristic feature of post-MI electrical instability, as it lowers myocardial tolerance to premature impulses and facilitates reentry formation [47]. Therefore, the observed recovery of VERP reflects an improvement in cardiomyocyte excitability and repolarization stability. Although moderate electrophysiological benefits were also observed in the PGO and CAM@Sal groups, the superior anti-arrhythmic efficacy of PGO/CAM@Sal suggests that its protective effects cannot be attributed to the improvement of a single electrical parameter, but rather arise from coordinated multi-level regulation.

Although in vivo electrophysiology reflected the overall electrical activity of the heart, it could not exclude the influence of blood flow, neural regulation, or other systemic factors. To precisely evaluate the intrinsic conduction and local abnormalities, ex vivo Langendorff-perfused hearts were examined using a multichannel mapping system (Fig. 6E). Additionally, 64-channel mapping revealed that conduction consistency was restored to varying degrees in all hydrogel-treated groups (Fig. 6F, Movie S1). PGO/CAM@Sal shortened conduction time (Fig. 6G), increased conduction velocity (Fig. 6H), and decreased heterogeneity (Fig. 6I), indicating a stabilized ventricular electrophysiology. These findings highlight the direct contribution of the conductive hydrogel framework to the reconstruction of myocardial electrical continuity at the tissue level.

To verify the relationship between improved conduction and enhanced cardiomyocyte coupling, Cx43 expressions were analyzed. Cx43 and cardiac troponin T (cTNT, cardiomyocyte marker) immunofluorescence (Fig. 6L) confirmed improved cardiomyocyte coupling, with PGO/CAM@Sal exhibiting the highest expression in the infarct and peri-infarct regions (Fig. 6J and K), supporting enhanced gap junction formation and electrical synchrony. Mechanistically, this improvement is not solely attributable to the intrinsic conductivity of the PPy-containing hydrogel, but is also closely associated with the suppression of inflammation and fibrosis, which otherwise disrupt gap junction integrity and cardiomyocyte alignment. Importantly, while the conductive network primarily facilitates electrical signal propagation and synchronization, the Sal-loaded microspheres play a complementary role by alleviating oxidative stress and inflammatory injury, thereby stabilizing the post-infarction microenvironment. Such microenvironmental modulation indirectly supports electrical remodeling by preserving viable myocardium, limiting fibrotic barriers, and maintaining gap junction architecture. Therefore, the superior therapeutic efficacy observed in the PGO/CAM@Sal group is best interpreted as the result of a synergistic interplay between electrical conduction restoration and pharmacological mitigation of pathological remodeling, rather than the dominance of either mechanism alone.

### 2.8 Antioxidant, anti-inflammatory, and pro-angiogenic effects of PGO/CAM@Sal hydrogel in vivo

During the initial three days after acute MI, the infarcted myocardium underwent severe oxidative stress and inflammatory infiltration, accelerating cardiomyocyte death and adverse ventricular remodeling. M2 macrophage polarization was initiated between days three and five, marking the transition to the reparative phase [14]. Therefore, infarcted myocardial tissues were harvested on day three post-surgery to assess the hydrogel-mediated regulation of inflammation and repair.

**Fig. 7:**
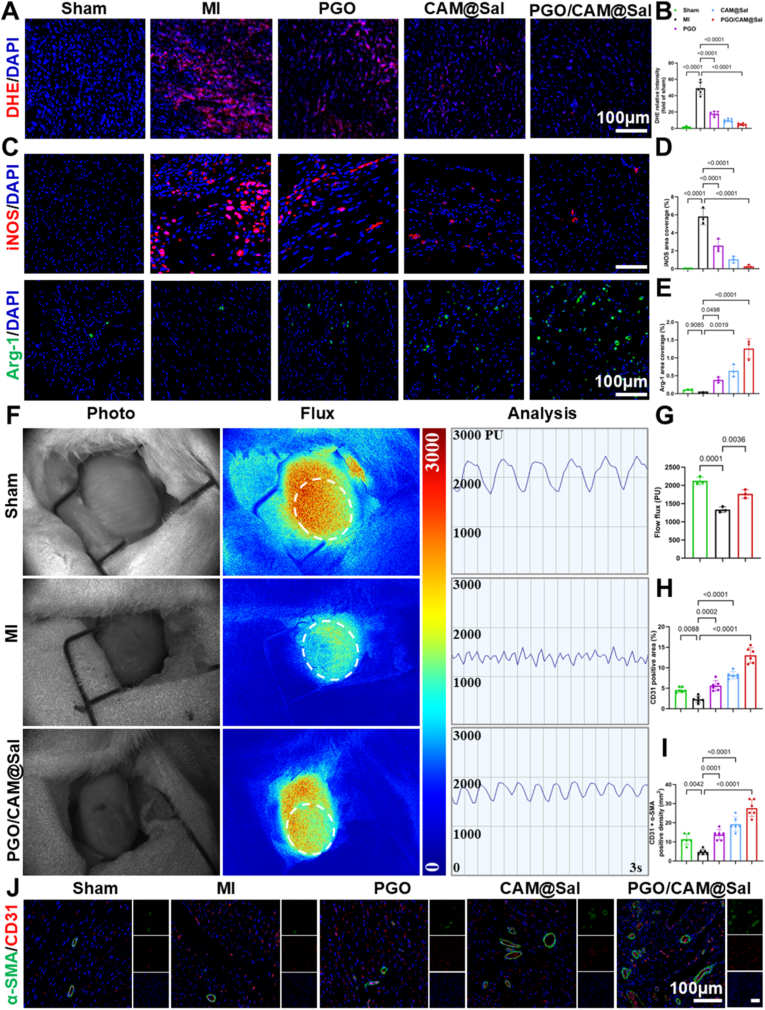
**Anti-oxidant, anti-inflammatory and pro-angiogenic effects of PGO/CAM@Sal Hydrogels in vivo.** A) Representative immunofluorescence images of DHE (red) staining in the infarcted myocardium at day 3 post-MI. B) Quantitative analysis of DHE fluorescence intensity in the infarcted regions across groups (n = 6). C–E) Representative images of iNOS (red) and Arg-1 (green) staining (C) with corresponding quantification (D–E) (n = 3). F) Representative images of the beating rat heart and corresponding full-field LSCI flux heat maps, along with blood perfusion curves of the infarcted regions analyzed by RFLSI Analysis software. All flux images were normalized using the same palette settings. G) Quantitative analysis of blood perfusion in each group by LSCI (n = 3). H–J) Representative immunofluorescence images of α-SMA (green) and CD31 (red) staining in the infarcted myocardium at day 28 post-MI (J) with corresponding quantification (H–I) (n = 6). (For interpretation of the references to color in this figure legend, the reader is referred to the Web version of this article.)

Dihydroethidium (DHE) staining (Fig. 7A) revealed a 49.06-fold increase in ROS generation for the MI group compared to that for the sham group (Fig. 7B), confirming elevated oxidative stress. Treatment with hydrogels markedly reduced ROS accumulation, with the PGO/CAM@Sal group exhibiting the lowest ROS intensity (only 4.92-fold greater than that of the sham group), demonstrating the synergistic antioxidant capacity of the boronate-ester network and Sal.

Effective cardiac repair required a dynamic shift in the macrophage phenotype from pro-inflammatory M1 to reparative M2 for attenuating chronic inflammation and fibrosis [33]. Immunostaining for iNOS and Arg-1 (Fig. 7C) demonstrated abundant M1 macrophages (5.80%) but scarce M2 macrophages (0.03%) in untreated MI hearts (Fig. 7D and E). Contrarily, PGO/CAM@Sal significantly suppressed M1 polarization (0.27%) and enhanced M2 polarization (1.26%) (Fig. 7D and E). M1 macrophages secreted pro-inflammatory cytokines such as IL-6 and TNF-α, which exacerbated tissue injury, whereas M2 macrophages released IL-10 to suppress residual inflammation and facilitate tissue repair and angiogenesis [48]. Consistent with the in vitro findings, immunohistochemistry analysis demonstrated reduced IL-6 and IL-1β expression and elevated IL-10 levels in PGO/CAM@Sal-treated hearts (Fig. S16).

In the chronic phase, ischemia-induced vascular rarefaction limited perfusion and exacerbated myocardial loss [49]. Laser speckle contrast imaging (LSCI) on day 28 (Fig. 7F) revealed that PGO/CAM@Sal treatment enhanced perfusion within the infarct zone by 32.31% relative to that in the MI group (Fig. 7G). Dual immunostaining of α-SMA (vascular smooth muscle marker) and CD31 (endothelial marker) (Fig. 7J) confirmed robust neovascularization, with 5.71-fold and 5.83-fold higher CD31^+^ and α-SMA^+^/CD31^+^ fluorescence intensities, respectively, than those for MI controls (Fig. 7H and I), suggesting formation of mature, functional vasculature.

Recent studies have extensively explored EGCG- and phenylboronic acid-functionalized hyaluronic acid-based hydrogels in inflammatory disease models, including postoperative abdominal adhesions [29,30], acute lung injury [31], and cardiac implant surface modification [50]. These systems consistently demonstrate that EGCG-based platforms constructed via dynamic boronate ester networks possess intrinsic antioxidant and anti-inflammatory properties. Notably, EGCG-containing coatings have also been reported to regulate cardiomyocyte apoptosis and metabolic signaling in cardiac repair settings [50].

In alignment with these established principles, the present hydrogel leverages EGCG–boronic chemistry to buffer oxidative stress and attenuate inflammatory cascades. Importantly, myocardial infarction involves temporally evolving oxidative burst, macrophage polarization, fibrotic remodeling, and electrophysiological disturbance. Therefore, beyond intrinsic antioxidative capacity, our system integrates stage-matched responsiveness, conductive PPy architecture, and sustained salidroside delivery to coordinate inflammatory regulation, oxidative balance, angiogenesis, fibrosis control, and electrical remodeling within infarcted myocardium. This integrative regulation across structural, molecular, and functional dimensions underlies the observed improvement in cardiac repair outcomes.

In summary, the PGO/CAM@Sal hydrogel effectively ameliorated the hostile postinfarction microenvironment through multiple mechanisms: (i) synergistic ROS scavenging and antioxidant protection, (ii) macrophage reprogramming toward the M2 phenotype with anti-inflammatory cytokine induction, and (iii) enhancement of tissue perfusion and angiogenesis. These integrative effects collectively contributed to structural preservation and functional cardiac recovery.

### 2.9 Potential mechanisms of PGO/CAM@Sal in cardiac repair and functional recovery

**Fig. 8:**
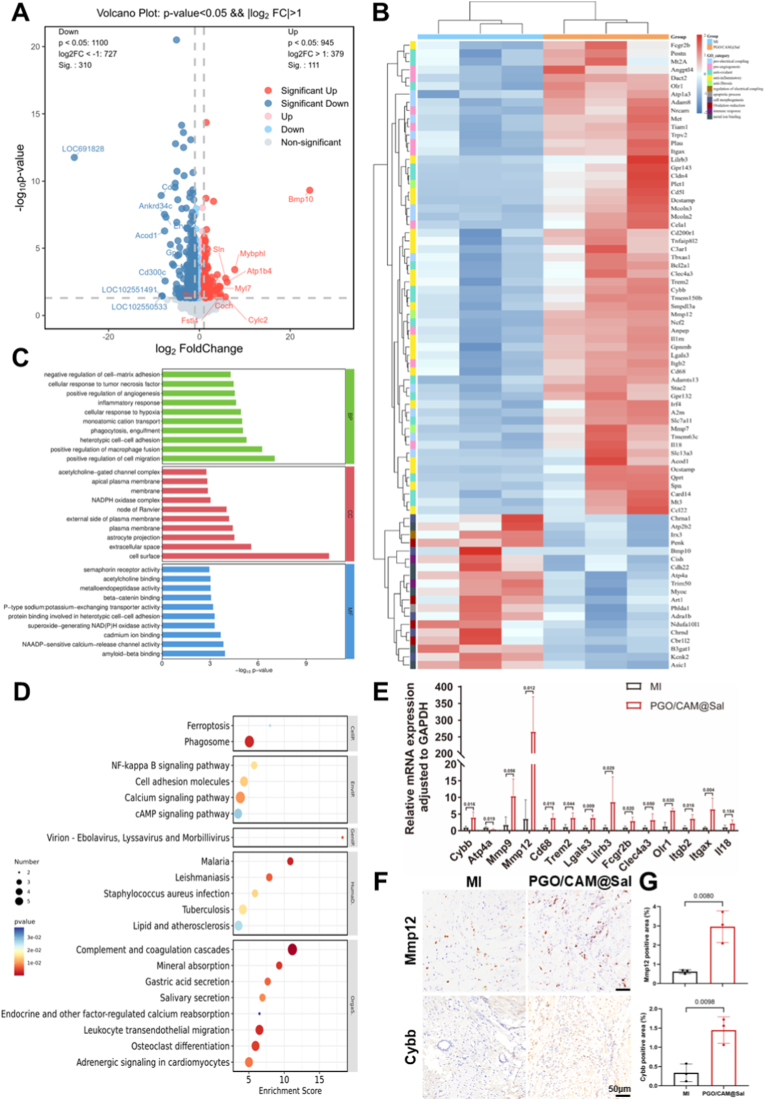
**Potential mechanisms of PGO/CAM@Sal in cardiac repair and functional recovery.** A) Volcano plot showing DEGs between MI and PGO/CAM@Sal-treated hearts. B) Heatmap of DEGs related to pro-electrical coupling, pro-angiogenesis, anti-oxidant, anti-inflammatory, and anti-fibrotic phenotypes (*p* < 0.05, |fold change| > 2). C) GO enrichment analysis of the top 30 DEGs (Total) (BP, biological process; CC, cellular component; MF, molecular function). D) KEGG pathway enrichment of the top 30 DEGs (Total) (CellP, cellular processes; EnvIP, environmental information processing; GenIP, genetic information processing; HumaD, human diseases; OrgaS, organismal systems). E) qRT-PCR validation of core and cluster-associated genes identified by transcriptomic and PPI analyses. F) Representative MMP12 and Cybb immunohistochemical staining in infarcted myocardium at 28 days post-MI. G) Quantitative analysis of MMP12 and Cybb (n = 3).

To elucidate the underlying molecular mechanisms, transcriptomic profiling of the infarcted myocardium was performed 28 days post-treatment. Compared to the MI group, PGO/CAM@Sal treatment resulted in 111 upregulated and 310 downregulated genes (*p* < 0.05, |fold change| > 2) (Fig. 8A). Heatmaps of the top 50 differentially expressed genes (DEGs) revealed distinct transcriptional landscapes between the groups (Fig. S17).

Focusing on key post-MI processes, including inflammation, oxidative stress, angiogenesis, fibrosis, and electrophysiological regulation, PGO/CAM@Sal substantially remodeled myocardial gene expression (Fig. 8B). Representative genes included Cd68, Trem2, and Lgals3 for anti-inflammatory regulation; Cybb, Slc7a11, and Mt3 for antioxidant response; Cela1, Nrcam, and Angptl4 for angiogenesis; MMP12 and MMP7 for anti-fibrotic modulation; and Slc13a3, Mcoln3, and Atp1a3 for electrical coupling. Gene ontology (GO) enrichment revealed involvement in biological processes such as inflammatory response, cellular response to hypoxia, macrophage fusion, positive regulation of angiogenesis, phagocytosis and engulfment, monatomic cation transport, and positive regulation of cell migration (Fig. 8C). Kyoto Encyclopedia of Genes and Genomes (KEGG) pathway analysis highlighted NF-κB signaling, calcium and cAMP signaling, and adrenergic signaling in cardiomyocytes (Fig. 8D). These findings indicated that PGO/CAM@Sal broadly modulated postinfarction molecular networks, alleviating oxidative stress, reprogramming macrophage function, promoting vascular regeneration, maintaining ECM homeostasis, and enhancing electrical conduction, thereby assisting comprehensive structural and functional cardiac repair.

Key regulatory genes were identified by integrating co-expression analysis of genes upregulated (Fig. S18) in the MI group compared to the PGO/CAM@Sal group with a STRING-based protein–protein interaction (PPI) network. Cybb and Atp4a initially emerged as central nodes demonstrating both transcriptional relevance and protein-level connectivity (Fig. S19A). Additional PPI modules were also identified, including macrophage activation–related genes (Cd68, Trem2, Lgals3, Lilrb3) and leukocyte recruitment–associated genes (Il18, Itgb2, Itgax), as well as ECM-remodeling enzymes (Mmp9, Mmp12). However, quantitative reverse transcription polymerase chain reaction (qRT-PCR) validation refined these candidate networks. Mmp9, Clec4a3, and Il18 showed no significant differential expression (*p* > 0.05) and were therefore excluded from downstream consideration, whereas Mmp12 exhibited the most robust and consistent regulation (Fig. 8E). Moreover, although Atp4a appeared as a computational hub, existing studies indicate that Atp4a encodes the gastric H^+^/K^+^-ATPase α-subunit predominantly expressed in parietal cells and has no established functional relevance in cardiac tissue [51,52]. Consequently, Atp4a was interpreted as a context-independent computational artifact rather than a biologically meaningful regulator in MI.

To further strengthen the molecular-level evidence supporting the transcriptomic findings, immunohistochemical (IHC) analyses of Mmp12 and Cybb were performed to validate their expression at the protein level (Fig. 8F). Compared with the MI group, the PGO/CAM@Sal-treated hearts exhibited markedly enhanced immunoreactivity for both proteins. Quantitative analysis (Fig. 8G) revealed that the Mmp12-positive area increased from 0.62% in the MI group to 2.96% in the PGO/CAM@Sal group, while Cybb-positive staining increased from 0.34% to 1.45%, respectively. These results are consistent with the transcriptomic and qRT-PCR, extending the validation of the Mmp12/Cybb axis from the transcriptional to the protein level.

After integrating transcriptomic profiling, protein–protein interaction analysis, qRT-PCR validation, and protein-level IHC evidence, Mmp12 and Cybb emerged as the most credible core regulators (Fig. S19B). Mmp12, a macrophage-derived elastase, plays a pivotal role in ECM turnover, fibrosis modulation, and tissue structural remodeling after MI [53]. Cybb (Nox2), a classical NADPH oxidase that is highly expressed in cardiomyocytes, endothelial cells, and immune cells, catalyzed ROS production and propagated inflammatory signaling [54,55]. Its dysregulation was associated with oxidative injury, immune infiltration, fibrosis, and arrhythmogenesis [56,57]. Importantly, the involvement of the Mmp12/Cybb-related pathways identified here is highly consistent with multiple independent functional outcomes observed in this study, including significant attenuation of oxidative stress, suppression of inflammatory responses, reduction of fibrotic remodeling, and improvement of electrophysiological reconstruction. This convergence of molecular, histological, and functional evidence provides system-level support for the participation of the Mmp12/Cybb axis in the material-mediated therapeutic effects of PGO/CAM@Sal.

Despite the combined evidence obtained from transcriptomic profiling, qRT-PCR validation, and protein-level IHC analyses, the present mechanistic interpretation of the Mmp12/Cybb axis remains primarily correlative. While these multi-layered datasets consistently associate the regulation of Mmp12 and Cybb with the observed attenuation of oxidative stress, inflammatory responses, fibrotic remodeling, and electrophysiological dysfunction, direct causality cannot be conclusively established based on the current experimental framework. Definitive mechanistic validation would require targeted molecular interventions, such as gene knockdown or overexpression strategies, to directly interrogate the functional roles of these regulators in myocardial repair. Such approaches, often involving gene-modified animal models, represent a complementary but distinct line of investigation and warrant dedicated exploration in future studies.

## 3 Conclusions

This study presents a PGO/CAM@Sal smart hydrogel that enables the pH/ROS/MMP9 triple-responsive staged on-demand release of Sal at different MI stages. In vitro and in vivo, the hydrogel synergistically exerts anti-inflammatory, antioxidant, pro-angiogenic, anti-fibrotic, and conduction-improving effects, thereby improving electrophysiological stability, structural remodeling, and cardiac functions. Transcriptomics analysis indicates a potential involvement of the Mmp12/Cybb axis in modulating inflammation, oxidative stress, fibrosis, and calcium signaling, revealing multi-pathway and multi-target mechanisms. Overall, the PGO/CAM@Sal hydrogel offers an efficient, controllable, and comprehensive strategy for post-MI precision therapy.

## 4 Experimental section

### 4.1 Materials

Gelatin (medium gel strength), 4-carboxyphenylboronic acid (CPBA, ≥97%), hyaluronic acid (HA, ≥97%), sodium alginate (≥98%), and N-hydroxysuccinimide (NHS, ≥98%) were sourced from Aladdin Reagent (Shanghai, China). Epigallocatechin gallate EGCG (99.75%) and Sal (99.90%) were purchased from MedChemExpress (New Jersey, USA). Silk cocoons were degummed and processed to obtain a regenerated silk fibroin solution following a previously reported method [58]. Pyrrole (≥99%) was procured from Adamas Reagent (Shanghai, China). Ferric chloride (FeCl_3_, ≥99.99%), sodium periodate (NaIO_4_, 99.5%), and ethylene glycol (98%) were purchased from Macklin Reagent (Shanghai, China). 2-Morpholinoethanesulfonic acid (MES, ≥99.5%) was obtained from Sigma–Aldrich (St. Louis, MO, USA), and all other chemical reagents were sourced from Aladdin Reagent (Shanghai, China). CCK-8, cytotoxicity assay kits, and DCFH-DA ROS detection kits were purchased from Beyotime Biotechnology Co., Ltd. (Shanghai, China). Enzyme-linked immunosorbent assay (ELISA) kits for IL-6, TNF-α, and IL-10 were obtained from Seville Biotechnology Co., Ltd. (Wuhan, China). H9c2, HUVECs, and RAW264.7 cell lines were sourced from the Chinese Academy of Sciences Cell Bank.

### 4.2 Synthesis of CAM@Sal

CAM@Sal was prepared using a microfluidic method followed by post-impregnation. Further, 2% (w/v) sodium alginate solution (2 g/100 mL) served as the dispersed phase, mineral oil containing 5% (w/v) Span 80 served as the continuous phase, and 2% (w/v) aqueous calcium chloride solution was used as the collecting and cross-linking phase. In a T-shaped microfluidic chip, the dispersed and continuous phases were introduced at flow rates of 1.0 and 450 μL min^−1^, respectively, producing uniform alginate droplets, which were cross-linked by Ca^2+^ to form hydrogel microspheres. After washing the microspheres to remove residual oil, they were immersed in a Sal solution (1 mg/mL) for 12 h for drug loading. Excess loading solution was removed by centrifugation (3000×*g*, 5 min), and the resulting CAM@Sal microspheres were stored at 4 °C in the dark until further use.

### 4.3 Preparation of PS

CPBA-NHS was synthesized by dissolving 4-carboxyphenylboronic acid (2 g, 12 mmol) and NHS (2 g, 17 mmol) in 20 mL of DMF, cooling the solution in an ice bath, and then adding EDC·HCl (3 g, 18 mmol) with continuous stirring overnight at room temperature. The reaction mixture was sequentially washed with 0.1 M HCl and 0.1 M NaHCO_3_, each in three 50 mL portions, dried over anhydrous MgSO_4_, and the solvent was evaporated under reduced pressure to obtain CPBA-NHS. CPBA-SF was prepared by adding 6 wt% SF solution dropwise into 0.1 M MES buffer (6 mL) at 4 °C, followed by CA-NHS (40 mg) dissolved in DMSO (1 mL). The mixture, with its pH adjusted to 7.0, was stirred at room temperature for 6 h. The product, PS, was then purified through dialysis and centrifugation to eliminate debris.

### 4.4 Preparation of GPE and OHA

GP was synthesized by incorporating 15% w/w pyrrole into a 2 wt% gelatin solution, initiating polymerization with FeCl_3_ under nitrogen at 4 °C for 24 h. The resulting product underwent dialysis and lyophilization to yield a solid form. Gelatin (1.0 g) was dissolved in 20 mL of 0.05 M MES buffer, followed by the addition of EGCG (204 mg) and EDC·HCl (173 mg). The solution was stirred at 27 °C for 3 h, then dialyzed and lyophilized to obtain a solid product. GP and GE were combined in equal volumes and lyophilized to produce GPE.

OHA was prepared according to a previously reported protocol [18]. In summary, 4.0 g of HA was dissolved in 100 mL of deionized water.After dissolving completely, 1.08 g of NaIO_4_ was added and stirred at room temperature for 24 h. Subsequently, 0.5 mL of ethylene glycol was introduced to halt the reaction, with stirring continuing for an additional 2 h at room temperature. The resulting solution was dialyzed and lyophilized to obtain the OHA.

### 4.5 Preparation of PGO/CAM@Sal

PS (3 wt%), GPE (8 wt%), and OHA (0.5 wt%) solutions (PGO) were mixed in a 2:1:3 vol ratio, and CAM@Sal microspheres were added to a final wet weight concentration of 10 mg/L, corresponding to 5% of the total hydrogel volume. The mixture was gently stirred at 50 rpm, room temperature, for 5 min to ensure uniform microsphere distribution. The mixture was incubated at 37 °C for 60 min to ensure full hydrogel formation. The PGO/CAM@Sal hydrogels were kept at 4 °C in darkness.

### 4.6 Characterization methods

#### 4.6.1 Scanning electron microscopy

Freshly prepared PGO and PGO/CAM@Sal hydrogels were cut into blocks (≈3 × 3 × 1 mm^3^), frozen in liquid nitrogen for 30 min, and freeze-dried for 24 h. Dried samples were mounted on conductive adhesive and sputter-coated with gold (10 mA, 45 s) using a Quorum SC7620 coater. The microstructures were imaged using SEM (Sigma 500, Zeiss, Germany).

#### 4.6.2 Fourier transform infrared spectrometry

Approximately 20 mg of dried hydrogel was pressed between two glass slides into a thin film. FTIR spectra were obtained with a Nicolet iS10 spectrometer (Thermo Fisher, USA) using the attenuated total reflectance method, covering a range of 4000–500 cm^−1^ at a resolution of 4 cm^−1^.

#### 4.6.3 In vitro drug release study of hydrogels

The drug-release characteristics of the CAM@Sal microsphere and PGO/CAM@Sal hydrogel were assessed under various stimuli. Hydrogel samples were incubated at 37 °C with gentle shaking in 3 mL of various solutions: phosphate-buffered saline (PBS, pH 7.4), acetate buffer (pH 5.0), PBS with 200 μM H_2_O_2_, PBS with 1 μg/mL MMP9, and a combination of triple stimuli (pH 5.0, 200 μM H_2_O_2_, and 1 μg/mL MMP9).At specified intervals, 1 mL of the release medium was removed and replenished with an equal volume of fresh PBS under identical conditions. The concentration of Sal in the collected medium was measured using a UV–Vis spectrophotometer at its characteristic wavelength, and the cumulative release was calculated. Each condition was tested in triplicate.

#### 4.6.4 Rheological properties of hydrogels

The rheological characteristics of the hydrogels were assessed at 37 °C using a HAAKE Rheostress RS6000 rheometer (Thermo Fisher, Germany). A 400 μL hydrogel sample was positioned between parallel plates with a 1 mm separation. Frequency sweep tests assessed the storage (G′) and loss (G″) moduli, whereas strain sweep tests identified the linear viscoelastic region and critical strain point. Shear-thinning behavior was observed. All measurements were performed in triplicate.

#### 4.6.5 Electrochemical performance of hydrogels

The electrochemical properties of the hydrogels were evaluated using two methods. The conductivity was measured using the four-point probe method (ST2922B, Lattice Electronics, China). Their electrochemical behavior was assessed using an electrochemical workstation (Autolab Vionic, Metrohm, Switzerland). A glassy carbon electrode coated with hydrogel served as the working electrode, a Hg/Hg_2_Cl_2_ electrode as the reference, and a platinum electrode as the counter electrode, with 0.1M PBS as the electrolyte. Cyclic voltammetry was performed from −0.6 to 0.8 V at a scan rate of 100 mV/s. All experiments were conducted in triplicate.

### 4.7 In vitro functional evaluation

#### 4.7.1 Antioxidant activity

The DCFH-DA assay was utilized to assess intracellular ROS levels. H9c2 cells (1 × 10^5^ cells/well) were seeded and cultured overnight. Cells were exposed to oxidative stress using 200 μM H_2_O_2_ for 24 h, then treated with hydrogel extracts (PGO, CAM@Sal, or PGO/CAM@Sal) for another 24 h. Subsequently, they were incubated with 20 μM DCFH-DA at 37 °C for 30 min, followed by fluorescence imaging with a fluorescence microscope.

#### 4.7.2 Macrophage polarization

Macrophage polarization was assessed using RAW264.7 cells. Cells were first exposed to 1 μg/mL LPS to trigger inflammation, followed by a 24-h co-incubation with hydrogel extracts. Immunofluorescence staining using iNOS and Arg-1 markers identified M1 and M2 macrophages, respectively.

#### 4.7.3 Enzyme-linked immunosorbent assay

Cytokine concentrations were quantified using a standard curve method via ELISA. RAW264.7 cells were exposed to hydrogel extracts for 72 h and subsequently rinsed with PBS. Cell lysis buffer was added to disrupt cell membranes and release the intracellular contents. Samples were obtained by centrifuging lysates at 4 °C and collecting the supernatants.IL-6, TNF-α, and IL-10 expression levels were measured using ELISA kits following the manufacturer's guidelines.

#### 4.7.4 In vitro OGD model

To simulate ischemic injury and evaluate the pro-electrical coupling effect of the conductive hydrogels, an in vitro OGD model was established using H9c2 cells. The cells were incubated in glucose-free DMEM and placed in an anaerobic chamber containing 95% N_2_ and 5% CO_2_ at 37 °C for 4 h [59]. Following OGD treatment, the cells were exposed to the hydrogel extract for 24 h and subsequently washed with PBS. Immunofluorescent staining of Cx43 (26980-1-AP, Proteintech) was performed to assess alterations in electrical coupling.

### 4.8 In vivo functional evaluation

#### 4.8.1 MI rat model and grouping

All animal experiments were approved by the institutional Animal Care and Use Committee. Sixty male Sprague–Dawley rats (200 ± 20 g) were obtained from Beijing Vital River Laboratory Animal Technology Co., Ltd. The experiments were conducted under sterile conditions. The rats were anesthetized with 2% isoflurane, intubated, mechanically ventilated, and continuously monitored via ECG. The heart was accessed via the fourth left intercostal space, and a 6-0 suture was used to ligate the left anterior descending coronary artery, inducing MI. Successful MI was indicated by a pale-gray anterior left ventricular wall near the apex and ST-segment elevation on the ECG. Immediately after MI induction, 100 μL of saline or hydrogel was injected into the infarct zone and two surrounding regions using a 30-G needle. Sham-operated rats experienced thoracotomy without the coronary ligation procedure. The chest was sutured, disinfected with iodine, and the rats were given unrestricted access to a standard pellet diet and water. The rats were randomly divided into five groups: Sham (n = 12, 100 μL saline), MI (n = 12, 100 μL saline), PGO (n = 12, 100 μL hydrogel), CAM@Sal (n = 12, 100 μL hydrogel containing 100 μg Sal), and PGO/CAM@Sal (n = 12, 100 μL hydrogel containing 100 μg Sal). To eliminate biases and ensure impartial analysis, all animal experiments were conducted by double-blinded operators.

#### 4.8.2 Echocardiography

To minimize bias, echocardiography was performed by an experienced technician who was blinded to the experimental groups. Left ventricular function was evaluated with a Vevo 2100 ultrasound system (VisualSonics, Canada) using an MS250 rat linear array transducer (13–24 MHz). Echocardiography was performed on isoflurane-anesthetized rats at 14 and 28 days after treatment. Key parameters such as EF, FS, LVIDs, and LVIDd were measured. Measurements were averaged across three consecutive cardiac cycles.

#### 4.8.3 Electrophysiological study in vivo

Referring to a previous protocol [60], programmed electrical stimulation was performed 28 days post-treatment to evaluate arrhythmia susceptibility, including VERP, duration, and inducibility of VAs. The rats were anesthetized with 2% pentobarbital sodium and mechanically ventilated. A 2.4F 10-pole catheter (Kexin Medical & Biological Technology Co., Ltd., Shanghai, China) was inserted into the right ventricle via the jugular vein, and the catheter tip was connected to a Lead-7000 electrophysiology recorder (Jinjiang Electronic Science and Technology Inc., Chengdu, China). VERP was determined using an S_1_–S_2_ pacing protocol: eight basic stimuli (S_1_) were applied, each followed by a premature stimulus (S_2_), with S_1_–S_2_ intervals decreased in 5-ms steps until failure of ventricular capture [61]. Burst pacing (50 ms cycle length, 10 s duration) was applied ten times at 30 s intervals to induce VAs. VAs were classified according to the Lambeth Convention as ventricular ectopic beats, couplets, triplets, ventricular tachycardia, and ventricular fibrillation [62]. The rats were euthanized humanely using an overdose of pentobarbital.

#### 4.8.4 Electrical mapping study ex vivo

Ex vivo electrical conduction in Langendorff-perfused hearts was assessed using an EMS64-USB-1003 mapping system and VCS-3001 stimulator (MappingLab, London, UK). A 64-channel multielectrode array (8 × 8) was positioned in the healthy myocardium or infarct border zone. Bipolar electrical stimulation was applied at the apex at 5 Hz with a current twice the threshold. EMapScope software was utilized to record local field potentials and activation times, enabling the creation of conduction heat maps and the calculation of conduction velocity.

#### 4.8.5 Laser speckle contrast imaging

Blood flow in the infarcted left ventricle was quantified using LSCI (RFLSI ZW, RWD Life Science, Shenzhen, China). After thoracotomy and heart exposure, the LSCI probe was placed on the cardiac surface. A 785 nm laser was used with a detection range of 0–3000 PU. Blood flow signals were recorded downstream of the left anterior descending coronary artery ligation, and tissue reflection images were obtained using a CCD. Pseudocolor perfusion maps were created for visualization, and diastolic blood flow intensity at each time point was assessed using RFLSI Analysis software.

### 4.9 Statistical analysis

All data were computed as mean ± standard deviation (SD). Statistical analyses were conducted with SPSS 20.0 (IBM, Armonk, NY, USA), while figures were created using Origin 2021 (OriginLab, Northampton, MA, USA) and GraphPad Prism 10 (GraphPad Software, San Diego, CA, USA). Two-tailed Student's *t*-tests were used to analyze statistical differences between the two groups. For comparisons among three or more groups, one-way ANOVA with Bonferroni post hoc tests or Kruskal–Wallis tests were used based on data distribution. A *p*-value <0.05 was deemed statistically significant.

## CRediT authorship contribution statement

**Jie Song:** Conceptualization, Investigation, Methodology, Writing – original draft. **Zhongxiong Fan:** Conceptualization, Investigation, Methodology, Writing – review & editing. **Yakun Bo:** Conceptualization, Methodology, Writing – original draft. **Dilare Taiwaikuli:** Formal analysis, Investigation, Methodology. **Jiayu He:** Data curation, Methodology, Software. **Xing Zhang:** Investigation, Methodology. **Shaokai Ji:** Data curation. **Yemin Chen:** Visualization. **Huanhuan Ding:** Formal analysis. **Heting Wu:** Conceptualization, Methodology, Writing – review & editing. **Chao Wang:** Funding acquisition, Software, Writing – review & editing. **Baopeng Tang:** Conceptualization, Project administration, Supervision. **Xianhui Zhou:** Conceptualization, Funding acquisition, Methodology, Project administration, Supervision, Writing – review & editing.

## Declaration of competing interest

The authors declare that they have no known competing financial interests or personal relationships that could have appeared to influence the work reported in this paper.

## Data Availability

Data will be made available on request.

## References

[1] Udell J.A., Bahit M.C., Campbell P. (2025). Prevention of heart failure after acute myocardial infarction. Lancet.

[2] Anderson J.L., Campion E.W., Morrow D.A. (2017). Acute myocardial infarction. N. Engl. J. Med..

[3] Yu C., Qiu Y., Yao F. (2024). Chemically programmed hydrogels for spatiotemporal modulation of the cardiac pathological microenvironment. Adv. Mater..

[4] Hu D., Li R., Li Y. (2024). Inflammation‐targeted nanomedicines alleviate oxidative stress and reprogram macrophages polarization for myocardial infarction treatment. Adv. Sci..

[5] Chen P., Zhang W., Fan X. (2024). A polyphenol-derived redox-active and conductive nanoparticle-reinforced hydrogel with wet adhesiveness for myocardial infarction repair by simultaneously stimulating anti-inflammation and calcium homeostasis pathways. Nano Today.

[6] Hohnloser S.H., Kuck K.H., Dorian P. (2004). Prophylactic use of an implantable cardioverter-defibrillator after acute myocardial infarction. N. Engl. J. Med..

[7] Hausenloy D.J., Botker H.E., Engstrom T. (2016). Targeting reperfusion injury in patients with ST-segment elevation myocardial infarction: trials and tribulations. Eur. Heart J..

[8] Bittle G.J., Morales D., Deatrick K.B. (2018). Stem cell therapy for hypoplastic left heart syndrome. Circ. Res..

[9] Cheng P., Cheng L., Han H. (2022). A pH/H_2_O_2_/MMP9 time‐response gel system with sparchigh tregs derived extracellular vesicles promote recovery after acute myocardial infarction. Adv. Healthcare Mater..

[10] Wagner M.J., Khan M., Mohsin S. (2020). Healing the broken heart; the immunomodulatory effects of stem cell therapy. Front. Immunol..

[11] Dixon J.A., Gorman R.C., Stroud R.E. (2009). Mesenchymal cell transplantation and myocardial remodeling after myocardial infarction. Circulation.

[12] Zhang L., Li T., Yu Y. (2023). An injectable conductive hydrogel restores electrical transmission at myocardial infarct site to preserve cardiac function and enhance repair. Bioact. Mater..

[13] Wang W., Tan B., Chen J. (2018). An injectable conductive hydrogel encapsulating plasmid DNA-eNOs and ADSCs for treating myocardial infarction. Biomaterials.

[14] Zhang L., Bei Z., Li T., Qian Z. (2023). An injectable conductive hydrogel with dual responsive release of rosmarinic acid improves cardiac function and promotes repair after myocardial infarction. Bioact. Mater..

[15] Wu Y., Chang T., Chen W. (2021). Release of VEGF and BMP9 from injectable alginate based composite hydrogel for treatment of myocardial infarction. Bioact. Mater..

[16] Qian B., Yang Q., Wang M. (2022). Encapsulation of lyophilized platelet-rich fibrin in alginate-hyaluronic acid hydrogel as a novel vascularized substitution for myocardial infarction. Bioact. Mater..

[17] Luo L., Li Y., Bao Z. (2023). Pericardial delivery of SDF‐1α puerarin hydrogel promotes heart repair and electrical coupling. Adv. Mater..

[18] Cheng N., Luo Q., Yang Y. (2025). Injectable pH responsive conductive hydrogel for intelligent delivery of metformin and exosomes to enhance cardiac repair after myocardial ischemia‐reperfusion injury. Adv. Sci..

[19] Wang L., Yu C., You T. (2025). Injection of ROS-responsive hydrogel loaded with IL-1β-targeted nanobody for ameliorating myocardial infarction. Bioact. Mater..

[20] Wei X., Chen S., Xie T. (2022). An MMP-degradable and conductive hydrogel to stabilize HIF-1α for recovering cardiac functions. Theranostics.

[21] Zhilan T., Zengyu Z., Pengpeng J. (2025). Salidroside promotes pro-angiogenesis and repair of blood brain barrier via Notch/ITGB1 signal path in CSVD model. J. Adv. Res..

[22] Zhou P., Ji J., Zhang Z. (2025). Salidroside-loaded metal-organic frameworks hydrogel to improve cardiac allograft function. J. Heart Lung Transplant..

[23] Chen H., Zhu J., Le Y. (2022). Salidroside inhibits doxorubicin-induced cardiomyopathy by modulating a ferroptosis-dependent pathway. Phytomedicine.

[24] Zhang Q., Wang Y.-Q., Li L. (2024). Fabrication and characterization of salidroside W/O/W emulsion with sodium alginate. Food Chem. X.

[25] Khan I.U., Shoukat M., Asif M. (2022). Assessing the synergistic activity of clarithromycin and therapeutic oils encapsulated in sodium alginate based floating microbeads. Microorganisms.

[26] Worke L.J., Barthold J.E., Seelbinder B. (2017). Densification of type I collagen matrices as a model for cardiac fibrosis. Adv. Healthcare Mater..

[27] Liang S., Zhang Y., Wang H. (2018). Paintable and rapidly bondable conductive hydrogels as therapeutic cardiac patches. Adv. Mater..

[28] Stumpf C., Seybold K., Petzi S. (2014). Interleukin‐10 improves left ventricular function in rats with heart failure subsequent to myocardial infarction. Eur. J. Heart Fail..

[29] Krishnan M., Alimi O., Kuss M. (2025). A dual-layer hydrogel barrier integrating bio-adhesive and anti-adhesive properties prevents postoperative abdominal adhesions. Adv. Healthcare Mater..

[30] Liu B., Kong Y., Alimi O. (2023). Multifunctional microgel-based cream hydrogels for postoperative abdominal adhesion prevention. ACS Nano.

[31] Liu B., Lush M., Nielsen M. (2025). Hyaluronic acid-based microgels loaded with fluticasone furoate for bleomycin-induced acute lung injury treatment. Mater. Today Bio.

[32] Wang X., Wang X., Tong M. (2016). SIRT6 protects cardiomyocytes against ischemia/reperfusion injury by augmenting FoxO3α-dependent antioxidant defense mechanisms. Basic Res. Cardiol..

[33] Zhou J., Liu W., Zhao X. (2021). Natural melanin/alginate hydrogels achieve cardiac repair through ROS scavenging and macrophage polarization. Adv. Sci..

[34] Chen H., Zhu J., Le Y. (2022). Salidroside inhibits doxorubicin-induced cardiomyopathy by modulating a ferroptosis-dependent pathway. Phytomedicine.

[35] Hou Y., Zhang Y., Jiang S. (2023). Salidroside intensifies mitochondrial function of CoCl_2_-damaged HT22 cells by stimulating PI3K-AKT-MAPK signaling pathway. Phytomedicine.

[36] Mihic A., Cui Z., Wu J. (2015). A conductive polymer hydrogel supports cell electrical signaling and improves cardiac function after implantation into myocardial infarct. Circulation.

[37] Paciello F., Pisani A., Rolesi R. (2024). Oxidative stress and inflammation cause auditory system damage via glial cell activation and dysregulated expression of gap junction proteins in an experimental model of styrene-induced oto/neurotoxicity. J. Neuroinflammation.

[38] Song Y., Zhang C., Zhang J. (2016). An injectable silk sericin hydrogel promotes cardiac functional recovery after ischemic myocardial infarction. Acta Biomater..

[39] Sun C., Wang Z., Zheng Q., Zhang H. (2012). Salidroside inhibits migration and invasion of human fibrosarcoma HT1080 cells. Phytomedicine.

[40] Li Y., Xue W., Li S. (2024). Salidroside promotes angiogenesis after cerebral ischemia in mice through Shh signaling pathway. Biomed. Pharmacother..

[41] Umadevi S., Gopi V., Elangovan V. (2014). Regulatory mechanism of gallic acid against advanced glycation end products induced cardiac remodeling in experimental rats. Chem. Biol. Interact..

[42] Liu Y., Zhou R., Guo Y. (2025). Muscle-derived small extracellular vesicles induce liver fibrosis during overtraining. Cell Metab..

[43] Zhao L., Liu H., Gao R. (2024). Brown adipose stem cell-loaded resilin elastic hydrogel rebuilds cardiac function after myocardial infarction via collagen I/III reorganisation. Gels.

[44] Mukherjee D., Sen S. (1993). Alteration of cardiac collagen phenotypes in hypertensive hypertrophy: role of blood pressure. J. Mol. Cellular Cardiol..

[45] Tallquist M., Molkentin J. (2017). Redefining the identity of cardiac fibroblasts. Nat. Rev. Cardiol..

[46] Zhang F., Zhang Y., Qian S. (2024). Injectable and conductive nanomicelle hydrogel with α-tocopherol encapsulation for enhanced myocardial infarction repair. ACS Nano.

[47] Kalla M., Hao G., Tapoulal N. (2020). The cardiac sympathetic co-transmitter neuropeptide Y is pro-arrhythmic following ST-elevation myocardial infarction despite beta-blockade. Eur. Heart J..

[48] Eming S.A., Wynn T.A., Martin P. (2017). Inflammation and metabolism in tissue repair and regeneration. Science.

[49] Wu X., Reboll M.R., Korf-Klingebiel M., Wollert K.C. (2021). Angiogenesis after acute myocardial infarction. Cardiovasc. Res..

[50] Wan H., Yang X., Zhang Y. (2024). Polyphenol-reinforced glycocalyx-like hydrogel coating induced myocardial regeneration and immunomodulation. ACS Nano.

[51] Krieg L., Milstein O., Krebs P. (2011). Mutation of the gastric hydrogen-potassium ATPase alpha subunit causes iron-deficiency anemia in mice. Blood.

[52] Read S., Hogan T.V., Zwar T.D., Gleeson P.A., Van Driel I.R. (2007). Prevention of autoimmune gastritis in mice requires extra-thymic T-cell deletion and suppression by regulatory T cells. Gastroenterology.

[53] Mouton A.J., Rivera Gonzalez O.J., Kaminski A.R. (2018). Matrix metalloproteinase-12 as an endogenous resolution promoting factor following myocardial infarction. Pharmacol. Res..

[54] Seimetz M., Sommer N., Bednorz M. (2020). NADPH oxidase subunit NOXO1 is a target for emphysema treatment in COPD. Nat. Metab..

[55] Emmerson A., Trevelin S.C., Mongue-Din H. (2018). Nox2 in regulatory T cells promotes angiotensin II–induced cardiovascular remodeling. J. Clin. Investig..

[56] Bache R.J., Chen Y. (2014). NOX2-induced myocardial fibrosis and diastolic dysfunction. J. Am. Coll. Cardiol..

[57] Prosser B.L., Ward C.W., Lederer W.J. (2011). X-ROS signaling: rapid mechano-chemo transduction in heart. Science.

[58] Di Buduo C.A., Soprano P.M., Tozzi L. (2017). Modular flow chamber for engineering bone marrow architecture and function. Biomaterials.

[59] Li H., Zhu J., Xu Y.-w. (2022). Notoginsenoside R1-loaded mesoporous silica nanoparticles targeting the site of injury through inflammatory cells improves heart repair after myocardial infarction. Redox Biol..

[60] Sun H., Song J., Li K. (2023). Increased β1-adrenergic receptor antibody confers a vulnerable substrate for atrial fibrillation via mediating Ca^2+^ mishandling and atrial fibrosis in active immunization rabbit models. Clin. Sci..

[61] Machhada A., Ang R., Ackland G.L. (2015). Control of ventricular excitability by neurons of the dorsal motor nucleus of the vagus nerve. Heart Rhythm.

[62] Walker M.J., Curtis M.J., Hearse D.J. (1988). The Lambeth Conventions: guidelines for the study of arrhythmias in ischaemia infarction, and reperfusion. Cardiovasc. Res..

