# Supplementary material for: Stage-matched conductive hydrogel with pH/ROS/MMP9 triple-responsive salidroside release for post-infarction myocardial repair
Source: Mater Today Bio. 2026 Mar 12;38:103016. doi: 10.1016/j.mtbio.2026.103016 (PMC13059318; doi:10.1016/j.mtbio.2026.103016)
Supplement: Multimedia component 1 [file mmc1.docx]

**Supporting Information**

**Stage-matched conductive hydrogel with pH/ROS/MMP9 triple-responsive salidroside release for post-infarction myocardial repair**

Jie Song ^a,b,1^, Zhongxiong Fan ^e,1^, Yakun Bo ^a,b,1^, Dilare Taiwaikuli ^a,b^, Jiayu He ^a,b^, Xing Zhang ^a,b^, Shaokai Ji ^c^, Yemin Chen ^a,b^, Huanhuan Ding ^a,b^, Heting Wu ^c,*^, Chao Wang ^c,*^, Baopeng Tang ^a,b,*^, Xianhui Zhou ^a,b,d,*^

^a^ *Department of Cardiac Pacing and Electrophysiology, The First Affiliated Hospital of Xinjiang Medical University, Urumqi, 830054, PR China*

^b^ *Xinjiang Key Laboratory of Cardiac Electrophysiology and Remodeling, The First Affiliated Hospital of Xinjiang Medical University, Urumqi, 830054, PR China*

^c^ *College of Pharmacy, Xinjiang Medical University, Urumqi, 830017, PR China*

^d^ *School of Public Health, Xinjiang Medical University, Urumqi, 830017, PR China*

^e^ *School of Pharmaceutical Sciences, Institute of Materia Medica, Xinjiang University, Urumqi, 830017, PR China*

^*^ Corresponding author.

*E-mail address:* [wuheting@xjmu.edu.cn](mailto:wuheting@xjmu.edu.cn) (H. Wu), [chaowang9999@outlook.com](mailto:chaowang9999@outlook.com) (C. Wang), [tangbaopeng1111@163.com](mailto:tangbaopeng1111@163.com) (B. Tang), [zhouxhuiyf@163.com](mailto:zhouxhuiyf@163.com) (X. Zhou).

^1^ These authors contributed equally to this work.

**1. Experimental Section**

*1.1. Particle size, zeta potential, and encapsulation efficiency*

CAM@Sal microspheres were dispersed in ultrapure water. Their particle sizes and zeta potentials were measured at room temperature using a Zetasizer Nano ZS90 (Malvern Instruments, UK). Each sample was analyzed in triplicate, and the mean values were reported.

To determine *encapsulation efficiency* (EE), the CAM@Sal microspheres were separated from the suspension, and the absorbance of the supernatant was recorded using a UV–Vis spectrophotometer (UV-2600i, Shimadzu, Japan). The concentration of free Sal in the supernatant was determined using a pre-established standard calibration curve. The EE was calculated as:

EE (%) = $\frac{\text{W}\text{total}-\text{W}\text{free}}{\text{W}\text{total}}$×100%

where W_total_ is the total amount of initially added Sal and W_free_ represents the amount of unencapsulated Sal in the supernatant.

*1.2. Stability assessment of CAM and CAM@Sal microbeads in buffer*

To evaluate the colloidal and morphological stability of CAM and CAM@Sal microbeads under aqueous conditions, microbeads were dispersed in PBS and incubated at 37 °C. At predetermined time points (0, 6, 12, and 24 h), the hydrodynamic diameter and zeta potential were measured using a Zetasizer Nano ZS90 (Malvern Instruments, UK). All measurements were performed in triplicate (n = 3) for each sample. The experimental results are summarized in **Table S1**.

**Table S1. Time-dependent colloidal stability of CAM and CAM@Sal microbeads in PBS (0–24 h)**

| **Sample** | **Time (h)** | **Diameter**  **(μm, mean ± SD)** | **Zeta potential (mV, mean ± SD)** | ***p* value vs 0 h (Diameter)** | ***p* value vs 0 h (Zeta)** |
| --- | --- | --- | --- | --- | --- |
| CAM | 0 | 104.58 ± 3.12 | −68.60 ± 0.97 | − | − |
|  | 6 | 105.31 ± 3.46 | −67.91 ± 1.12 | 0.42 | 0.31 |
|  | 12 | 103.87 ± 3.28 | −69.42 ± 1.05 | 0.51 | 0.27 |
| **Sample** | **Time (h)** | **Diameter**  **(μm, mean ± SD)** | **Zeta potential (mV, mean ± SD)** | ***p* value vs 0 h (Diameter)** | ***p* value vs 0 h (Zeta)** |
|  | 24 | 105.92 ± 3.67 | −68.13 ± 1.18 | 0.33 | 0.44 |
| CAM@Sal | 0 | 118.82 ± 3.56 | −58.57 ± 0.85 | − | − |
|  | 6 | 119.74 ± 3.91 | −57.94 ± 0.96 | 0.39 | 0.34 |
|  | 12 | 117.96 ± 3.62 | −59.21 ± 0.88 | 0.47 | 0.29 |
|  | 24 | 120.58 ± 4.05 | −58.36 ± 1.02 | 0.28 | 0.48 |

*1.3. Optimization and selection of PS: GPE:OHA ratio*

In the PGO hydrogel formulation, PS (3 wt%), GPE (8 wt%), and OHA (0.5 wt%) solutions were mixed at varying volume ratios to optimize hydrogel performance. The tested ratios included 2:1:1, 2:1:2, 2:1:3, and 2:1:4. During optimization, the GPE ratio was fixed as the primary conductive component, while the PS and OHA ratios were systematically adjusted to evaluate their effects on gelation behavior, mechanical properties, and electrical conductivity.

For each formulation, three independent hydrogel samples (n = 3) were prepared. The mixtures were gently stirred at room temperature for 5 min, followed by incubation at 37 °C to complete gelation. Gelation time, electrical conductivity, and compressive modulus were measured according to standard protocols. The experimental results are summarized in **Table S2**.

**Table S2. Properties of PGO Hydrogels at Different PS: GPE: OHA Ratios**

| **PS:GPE:OHA ratio** | **Gelation behavior**  **(time)** | **Electrical conductivity (S/cm)** | **Compressive modulus (kPa)** |
| --- | --- | --- | --- |
| 1:1:1 | No gelation | **−** | **−** |
| 2:1:1 | Extremely slow  (~60 min) | (4.13 ± 0.21) ×10⁻^4^ | 2.42 ± 0.27 |
| 2:1:2 | Slow (~15 min) | (6.12 ± 0.41) ×10⁻^4^ | 8.32 ± 0.76 |
| 2:1:3 | Fast (~30 s) | (7.35 ± 0.33) ×10⁻^4^ | 22.39 ± 0.90 |
| 2:1:4 | Extremely fast (~5 s) | (7.82 ± 0.28) ×10⁻^4^ | 35.64 ± 1.21 |

*1.4. Equilibrium swelling test of hydrogels*

Lyophilized hydrogels were dried at 40 °C to a constant weight and recorded as W_0_. Then, the samples were immersed in 0.01 M phosphate-buffered saline (PBS, pH 7.4) at 37 °C under gentle shaking. At predetermined time intervals (10–100 h), the hydrogels were removed, gently blotted with a filter paper to eliminate surface water, and weighed (W_t_). All experiments were conducted in triplicate. The swelling ratio (SR) was calculated as:

SR (%) = $\frac{\text{W}\text{t}-\text{W}\text{0}}{\text{W}\text{t}}$×100%

where W_0_ and W_t_ represent the weights of the swollen and dry hydrogels, respectively.

*1.5. Mechanical properties of hydrogels*

The compressive modulus of the hydrogels was measured using a universal testing machine (UTM 2103; Suns, China). Cylindrical hydrogel samples (approximately 10 mm height × 12 mm diameter) were compressed at a constant rate of 5 mm/min at room temperature. Stress–strain curves were recorded, and the compressive modulus was determined from the linear region. Each sample group was tested in triplicate.

*1.6 In vivo fluorescence imaging*

For in vivo tracking of hydrogel retention and degradation, the hydrogels were fluorescently labeled with Cy5.5 (HY-D0924, MCE) prior to administration. Briefly, Cy5.5 was conjugated to the hydrogel matrix and uniformly incorporated into CAM@Sal, PGO, and PGO/CAM@Sal formulations. Following left anterior descending coronary artery ligation, the labeled formulations were intramyocardially injected into four predefined sites within the peri-infarct border zone.

The in vivo distribution, retention, and degradation behavior of the hydrogels were systematically monitored using an in vivo imaging system (IVIS, PerkinElmer) at predetermined time points on Days 1, 3, 7, 14, and 21 post-injection. Fluorescence signals were acquired under identical imaging parameters, and quantitative analysis of signal intensity was performed using Living Image software (PerkinElmer, version 4.4) to assess temporal changes in hydrogel retention.

*1.7. Live/dead cell staining*

Cell viability was assessed using live/dead staining. H9c2 and HUVECs were seeded in 96-well plates and cultured for 24 h at 37 °C in a humidified 5% CO₂ atmosphere. Subsequently, the medium was replaced with 100 μL of hydrogel extracts (PGO, CAM@Sal, or PGO/CAM@Sal). After incubating for 24, 48, or 72 h, cells were washed with PBS and stained with 100μL of Calcein-AM/PI solution (Beyotime, China) for 10min at 37 °C in the dark. Fluorescent images were acquired using a fluorescence microscope (Olympus, Tokyo, Japan).

*1.8. In vitro cell viability*

Cell viability was quantified using a CCK-8 assay. H9c2 and HUVECs were seeded and treated with hydrogel extracts. After 24, 48, or 72 h, the medium was replaced with 20 μL of CCK-8 solution per well and incubated for 2 h. Absorbance at 450 nm was measured using a microplate reader.

*1.9. Hemolysis test*

Fresh rat blood was centrifuged at 3000 rpm for 10 min at 4 °C, and red blood cells (RBCs) were collected and washed three times with saline. RBCs (0.5 mL) were diluted with 9.5 mL of saline to prepare a 5% (v/v) suspension. For the negative control, 2 mL RBCs suspension was mixed with 100 μL saline; for the positive control, 2 mL RBCs suspension was mixed with 5 μL Triton X-100 (Solarbio, China) and 95 μL saline; and for the experimental groups, 2 mL RBCs suspension was mixed with 100 μL hydrogel extracts (PGO, CAM@Sal, or PGO/CAM@Sal). Samples were incubated at 37 °C for 3 h, centrifuged, and supernatants were photographed. Hemoglobin release was quantified by measuring the absorbance at 545 nm.

*1.10. Wound scratch assay*

HUVECs were seeded into 12-well plates and cultured for 24 h. A scratch was induced using a pipette tip, and the cells were washed twice with PBS. Hydrogel extracts were prepared by incubating hydrogels in high-glucose DMEM (pH 7.4) at 37 °C for 24 h. The extracts were then applied to HUVECs. Scratch closure was imaged at 0, 24, and 48 h, and the migration rate was computed as the ratio of the healed area to the initial scratch area.

*1.11. Tube formation assay*

Matrigel (50 μL) was added to pre-chilled 96-well plates and incubated at 37 °C for 30 min. HUVECs suspended in 100 μL medium were seeded onto Matrigel and treated with either culture medium, hydrogel extracts (PGO, CAM@Sal, or PGO/CAM@Sal). After 4 h, tube formation was imaged microscopically, and nodes, junctions, and branches were quantified using ImageJ software (v1.54g, NIH, USA).

*1.12. Histology and immunohistochemistry*

The rats from different treatment groups (Sham, MI, PGO, CAM@Sal, and PGO/CAM@Sal) were euthanized at 3 and 28 days post-MI. The heart was excised, rinsed thoroughly with PBS to remove residual blood, fixed in paraformaldehyde, and embedded in paraffin for sectioning. Systemic inflammatory status was assessed via whole blood analysis, while immunohistochemical staining of IL-6 (GB11117-100, Servicebio), TNF-α (GB115701-50, Servicebio), and IL-10 (GB11108-100, Servicebio) was used to evaluate inflammatory cell infiltration. Hematoxylin and eosin (H&E) staining was used to assess inflammation in major organs, and Masson’s trichrome staining was used to evaluate myocardial morphological changes and fibrosis. WGA staining was used to measure the cardiomyocyte cross-sectional area and type I and III collagen staining was used to analyze collagen deposition in the infarcted region. Myocardial functional markers were assessed by double labeling with Cx43 (26980-1-AP, Proteintech) and cardiac cTnT (ab8295, Abcam). ROS levels in the infarct zone were detected using DHE staining. Macrophage phenotypes were identified by iNOS (PA1-036, Invitrogen) and Arg-1 (GB15285-50, Servicebio) staining. Angiogenesis and vessel maturation were evaluated using double immunostaining for CD31 (ab222783, Abcam) and α-SMA (F3777, Merck). Immunohistochemical staining was performed to detect Mmp12 (22989-1-AP, Proteintech) and Cybb (GB11391-50, Servicebio), in order to further validate the transcriptomic findings at the protein level. All immunohistochemical, fluorescent, and morphological images were quantified using the ImageJ software (NIH, Bethesda, MD, USA).

*1.13. RNA sequencing analysis*

Total RNA was isolated from the infarcted left ventricular tissue of rats in the MI and PGO/CAM@Sal groups using the TRIzol reagent (Invitrogen, CA, USA). The RNA purity and integrity were assessed using a NanoDrop 2000 spectrophotometer (Thermo Scientific, USA) and an Agilent 2100 Bioanalyzer (Agilent Technologies, USA). RNA-seq libraries were prepared from 1 μg of total RNA using the VAHTS Universal V6 RNA-seq Library Prep Kit (Vazyme, China), and sequencing was performed on the Illumina Novaseq 6000 platform by OE Biotech Co., Ltd. (Shanghai, China). Raw reads were quality-controlled using FASTP to obtain clean reads, which were aligned to the reference genome using HISAT2. Gene expression levels were quantified as FPKM, and DEGs were identified using DESeq2 (q < 0.05, |fold change| > 2). Alternative splicing events were quantified using the percentage spliced-in (PSI) index and statistically analyzed using one-way analysis of variance (ANOVA).

*1.14. Quantitative reverse transcription polymerase chain reaction (qRT-PCR) analysis*

Total RNA from the MI region in different groups was extracted using the TRIzol method. RNA from each sample was reverse-transcribed into cDNA using the Reverse Transcription kit (AT341-02, TransGen). The qRT-PCR was performed with the SYBR® Premix ExTaq™ II Kit (Q311-03, Vazyme). Primers were designed based on the cDNA sequence in NCBI. GAPDH mRNA was used as an internal reference for detection. The primer sequences are listed in **Table S3**. The qRT-PCR reaction system includes: 5 μL of SYBR qRT-PCR master mix (2×), 0.2 μL of forward primer (10 μM), 1 μL of cDNA, and 3.6 μL of nuclease-free H_2_O. The total reaction system is 10 μL. Reaction conditions: 94 ℃ for 30 s, 94 ℃ for 3 s, 60 ℃ for 30 s. 45 amplification cycles were performed. The PCR was confirmed by the product melting curve, and the Ct values of the target gene and GAPDH of the sample were obtained based on the PCR curve. The relative expression level for target genes were performed with a value of 2^-ΔΔCt^.

**Table S3. Sequence of Primers for qRT-PCR**

| **Genes** | **Forward primer** | **Reverse primer** |
| --- | --- | --- |
| GAPDH | GCCTTCTCTTGTGACAAAGT | CTTGCCGTGGGTAGAGTCATA |
| Cybb | GGAATATTAGACTGATTGGAGGC | TCCAGCTTACAGAGTAGACGA |
| Atp4a | ATCTTGCTGGATGACAACTT | GGAATGTTCTTGGTCAGTGTATAG |
| Mmp9 | CTACTGCTGGTCCTTCTGAG | CTGGATATCAGCAATGGCAT |
| Mmp12 | TACACAGCATTCAGTCCCTCTA | CACAGTTGATGGTGGACTTC |
| Cd68 | GACAGCTTACCTTTGGATTCA | GTATTCCACTGCCATGTAGTTA |
| Trem2 | TCCTCCTGAGCAAGCTTCTTA | GTGAGGATCTGAAGTTGGTG |
| Lgals3 | CACTGACAGTGCCCTACGAT | CTTCACTGTGCCTATGATTGT |
| Lilrb3 | GATCAGTTCCTCTTCCTCAG | AAGTGTATGGTCTACACAGG |
| Fcgr2b | GTTAAGAGACTACAGCAACG | TAGTTGATCCTCGCTGAGATT |
| Clec4a3 | GGATTCACTCTCGGAAGACA | CAAGATTCTACTGAGTCAGTGG |
| Olr1 | TGGATGGGATTACATCGGAA | GTGCCTGATGAGTACATCTG |
| Itgb2 | CATTGATCTGTACTACCTCATGG | GGACTCTGTGATCTCGTTGA |
| Itgax | TACTCTGCGTGCTACAAGAA | CATTTGTGCCACTGTTAAGG |
| Il18 | AACAGCAGCCAATGTTCTTA | TAACGTGGAGCATAGAGCAA |

**2. Supplementary Figures**

**
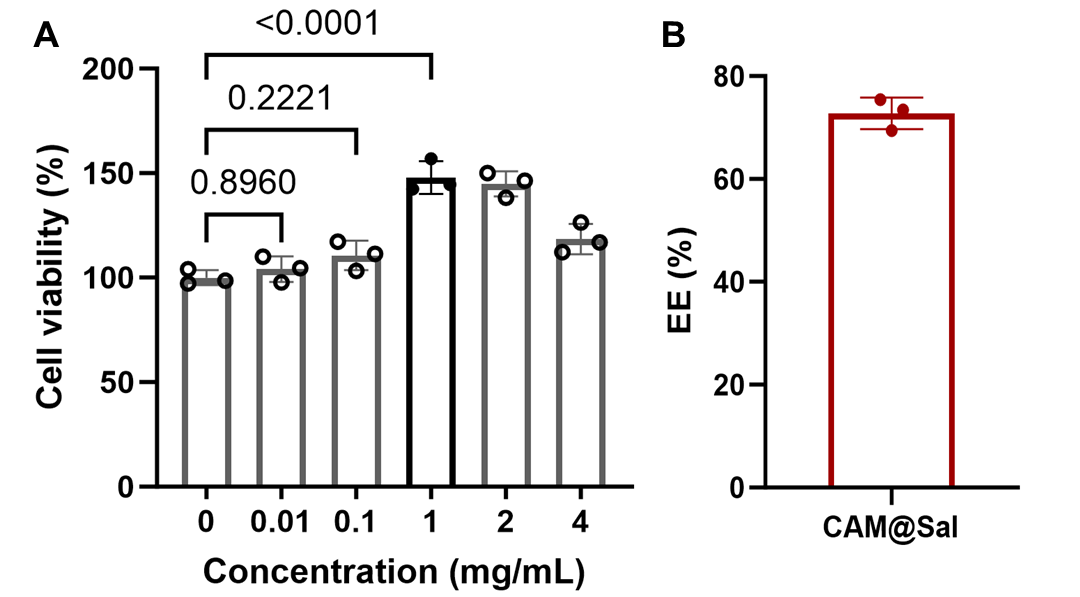
**

**Figure S1.** A) CCK-8 assay of H9c2 cell viability at different salidroside concentrations (n = 3). B) Encapsulation efficiency (EE) of CAM@Sal (n = 3).


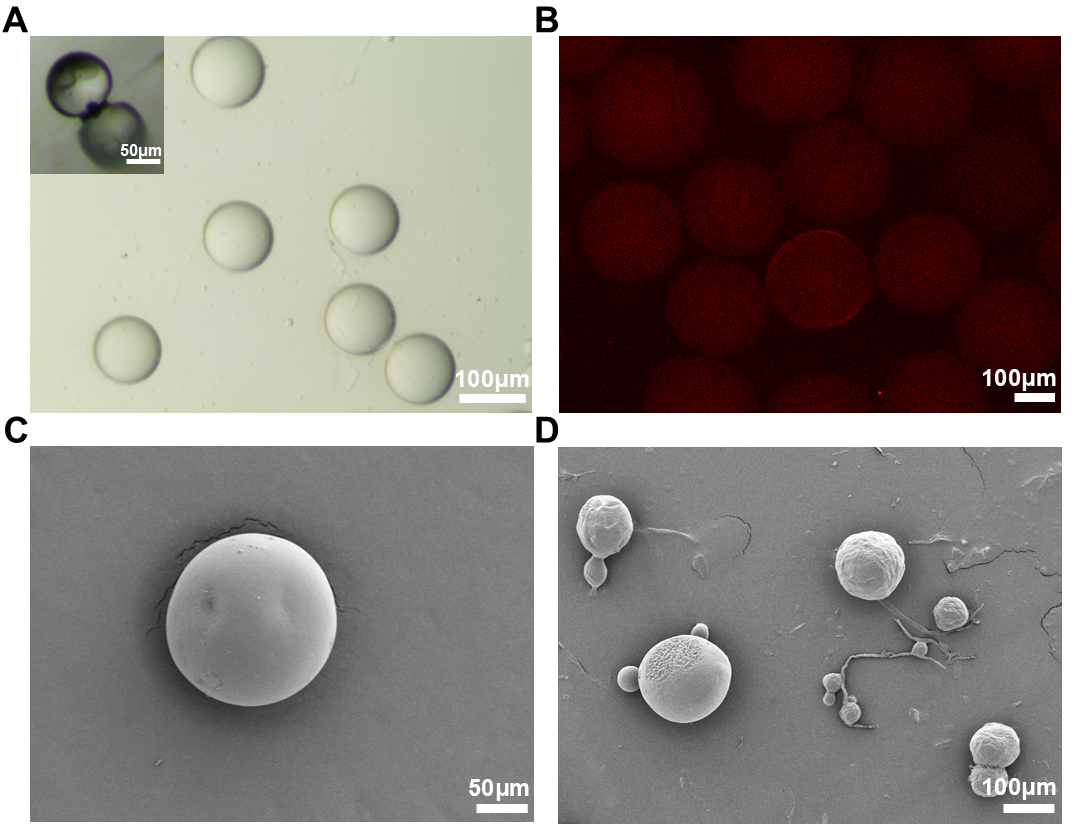


**Figure S2.** A) Bright-field microscopy image of CAM. B) Confocal laser scanning microscopy image of CAM. C) SEM image of CAM. D) SEM images of CAM@Sal.


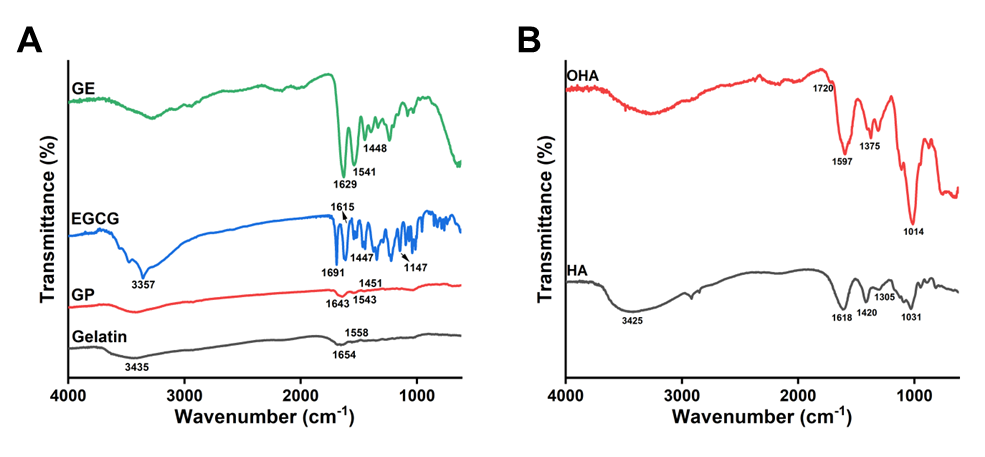


**Figure S3.** FTIR characterization of composite components in the PGO hydrogel system. A) FTIR of EGCG , GP and GE. B) FTIR of HA and OHA.


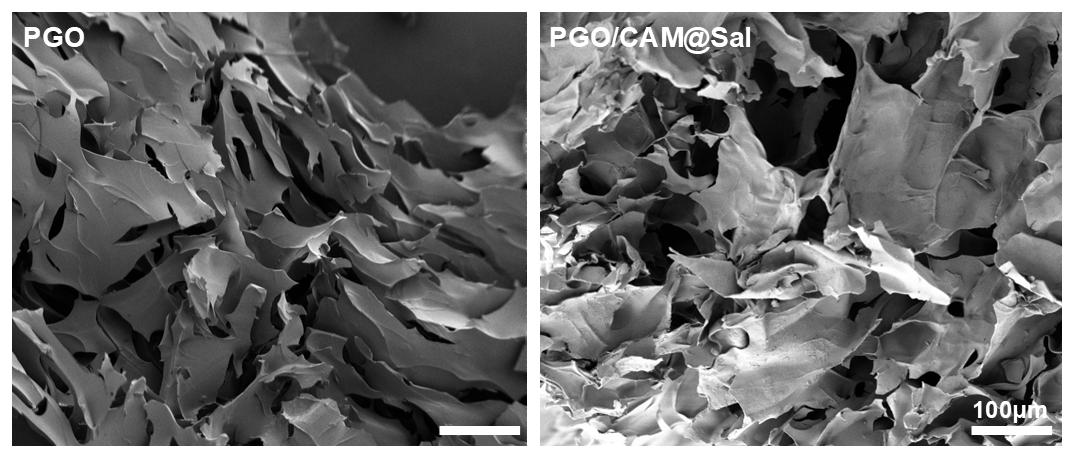


**Figure S4.** SEM images of PGO and PGO/CAM@Sal hydrogels after 14 days of degradation.


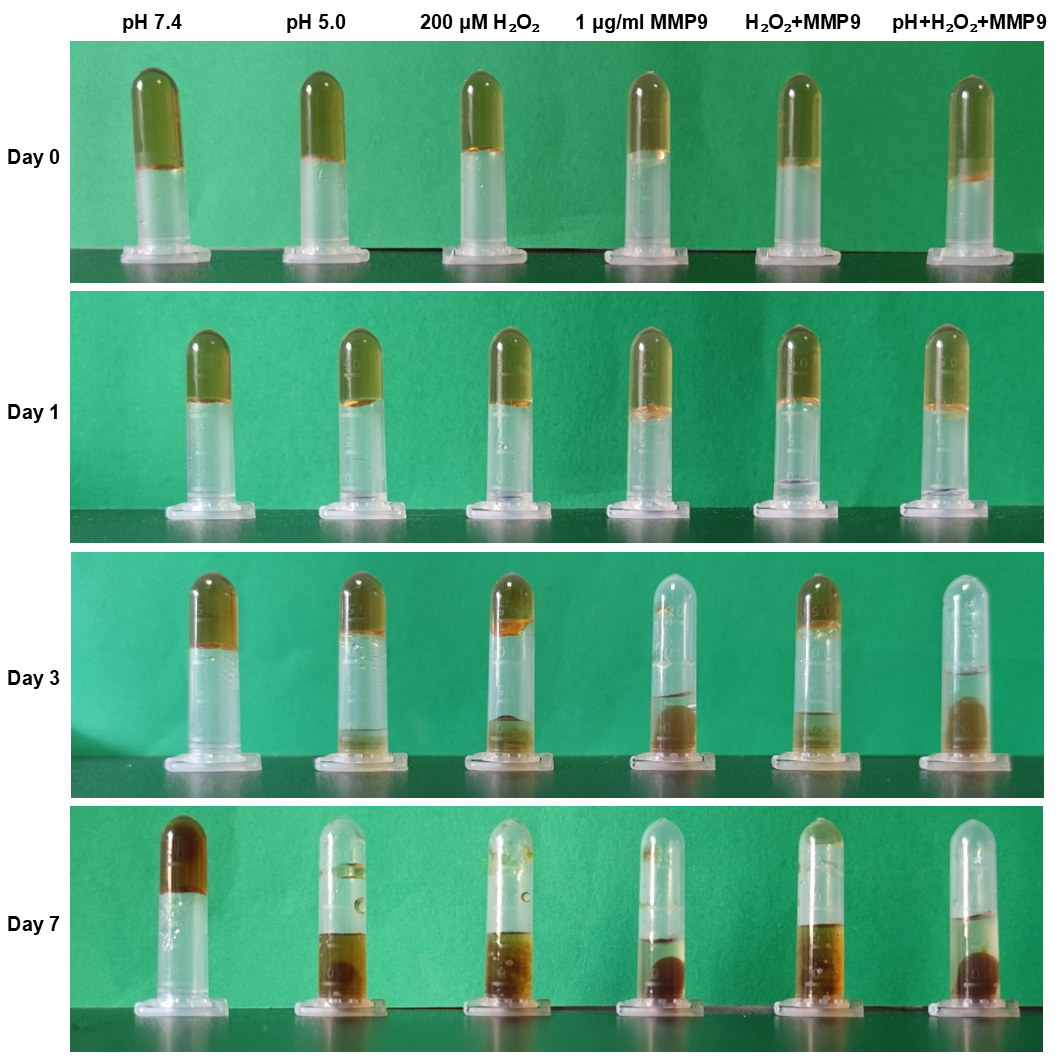


**Figure S5.** Representative photographs of PGO/CAM@Sal hydrogels under different conditions.


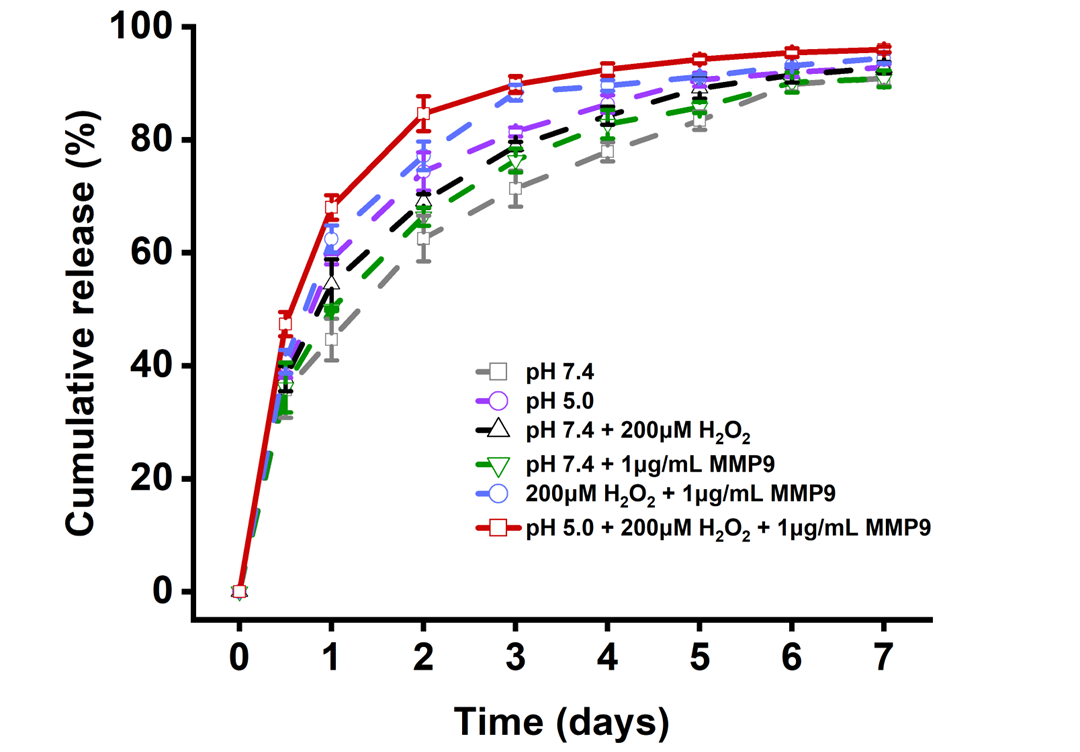


**Figure S6.** Release profiles of Sal from CAM@Sal microsphere under different conditions (n = 3).


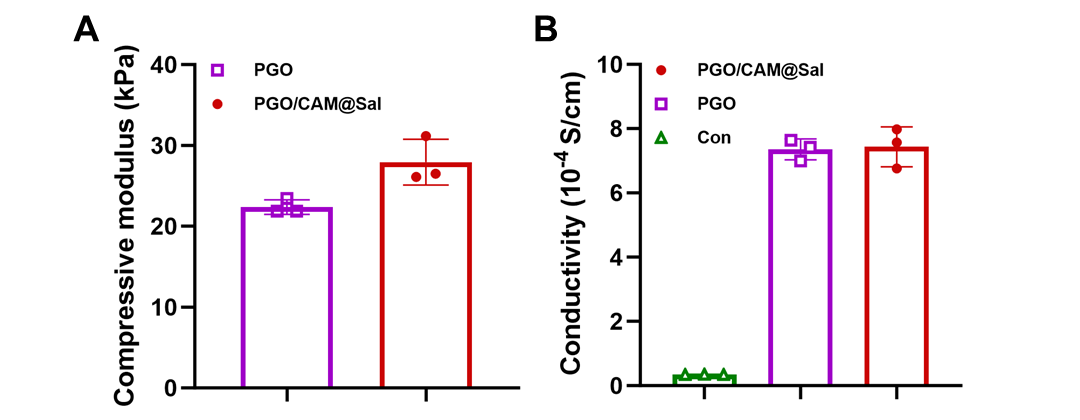


**Figure S7.** Compressive modulus (A) and conductivity (B) of different hydrogels (n = 3).


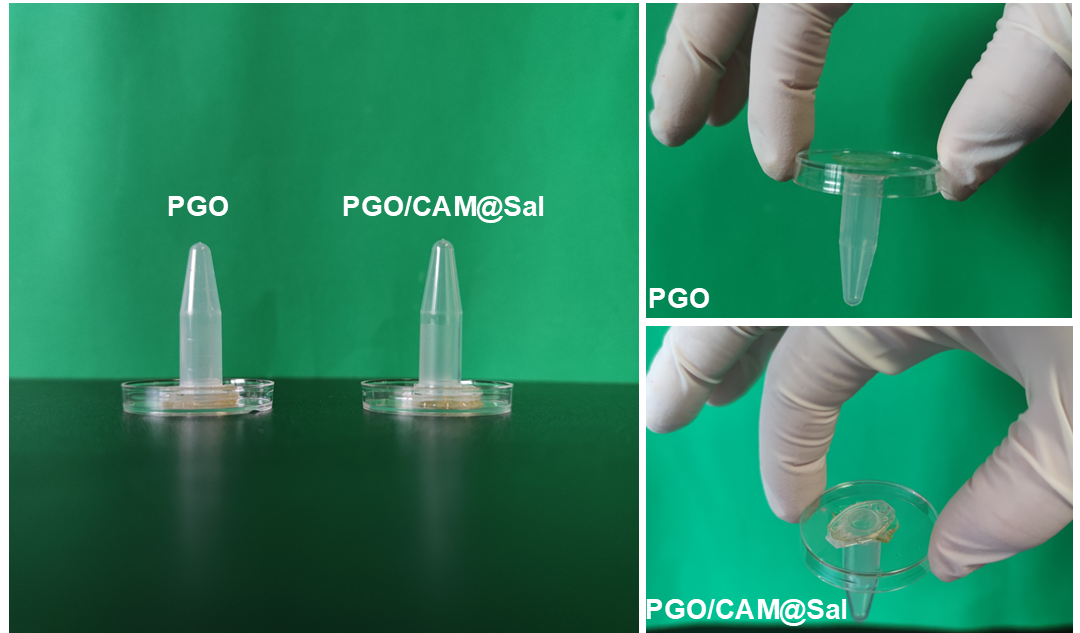


**Figure S8.** Characterization of adhesion performance of hydrogels to inner wall of centrifuge tubes.


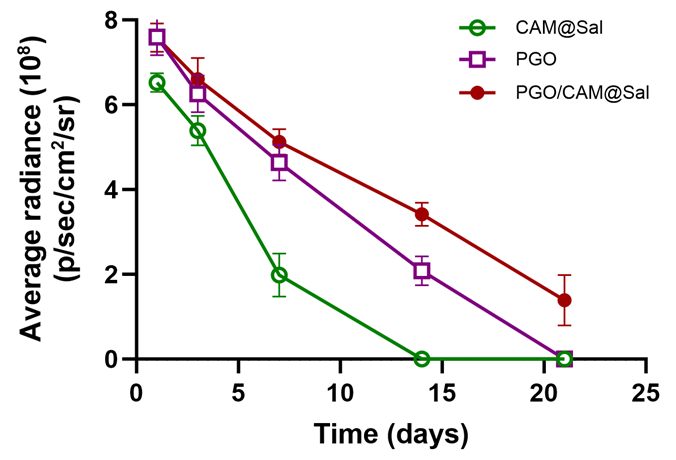


**Figure S9.** Quantification of average radiance from IVIS fluorescence imaging in rats treated with different hydrogels (n=3).


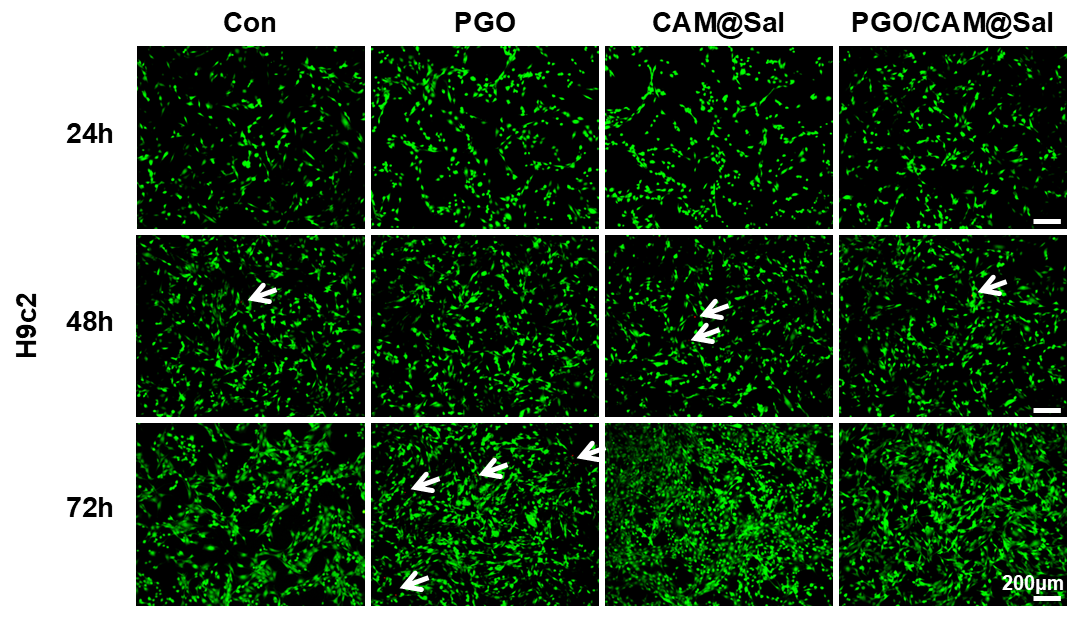


**Figure S10.** Live/dead staining of H9c2 after treatment with hydrogel extracts (green: live cells; red: dead cells).


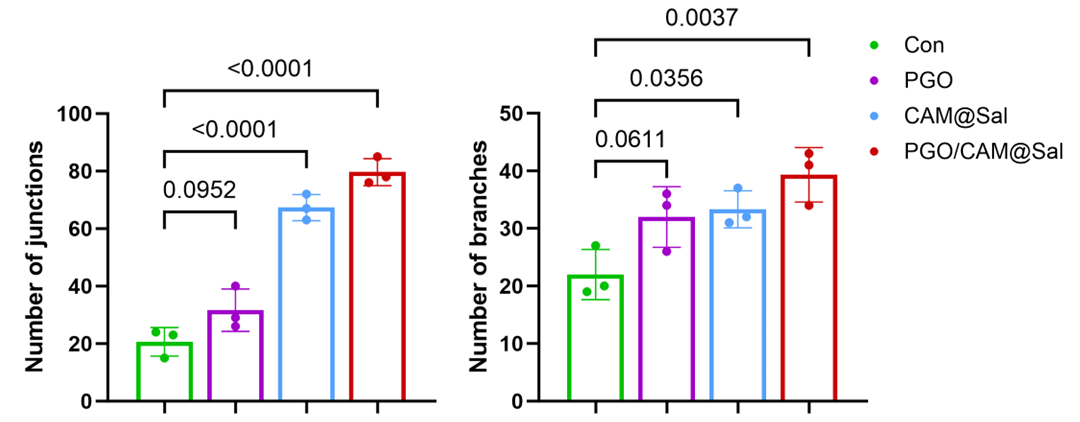


**Figure S11.** Quantitative analysis of junction numbers and branch numbers among different hydrogel groups (n = 3).


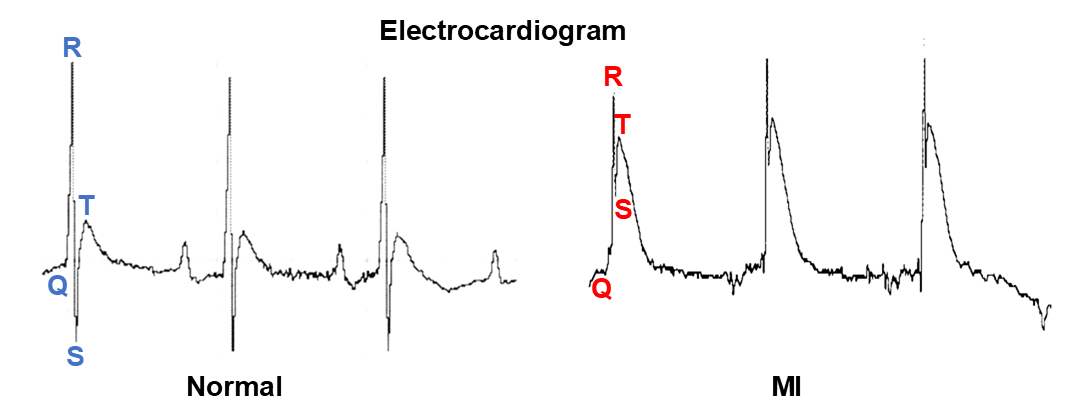


**Figure S12.** The MI model was confirmed by electrocardiogram.


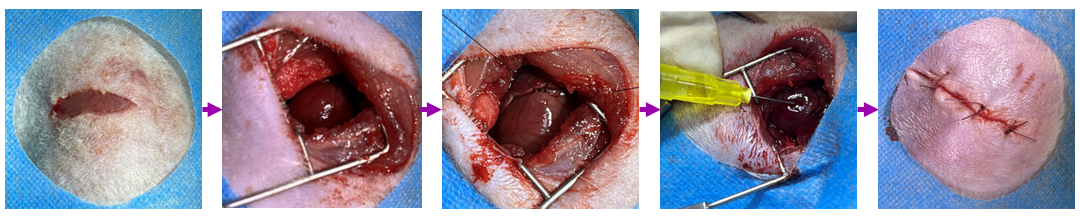


**Figure S13.** Schematic diagram of intramyocardial in situ hydrogel injection in MI rat model.


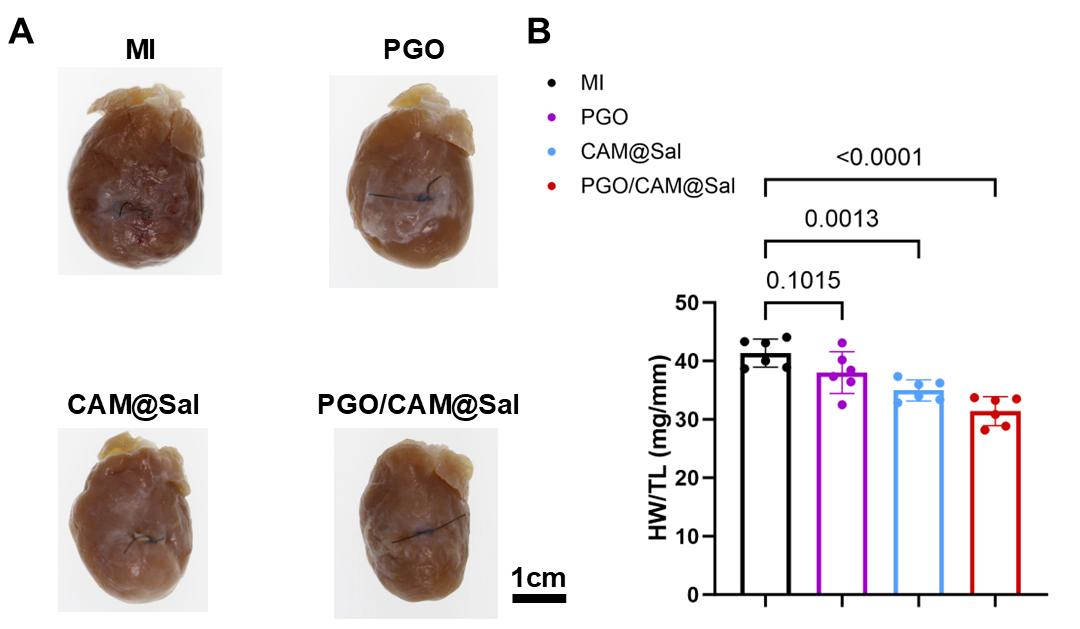


**Figure S14.** A) Representative cardiac morphology in rats from different groups at 28 days post-MI. B) Heart weight/tibia length (HW/TL) ratios in each group (n = 6).


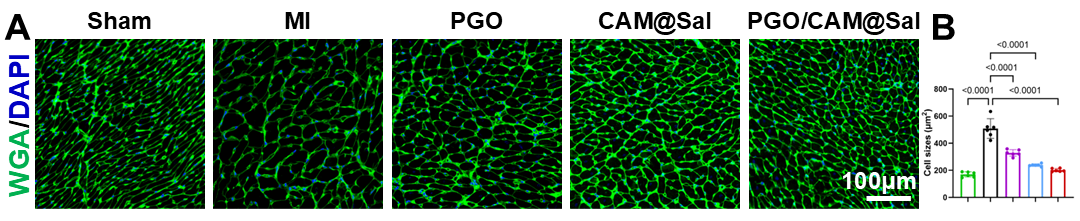


**Figure S15.** A) Representative wheat-germ agglutinin (WGA) staining of myocardial sections from different groups at 28 days post-MI. B) Quantification of cardiomyocyte cross-sectional area in each group (n = 6).


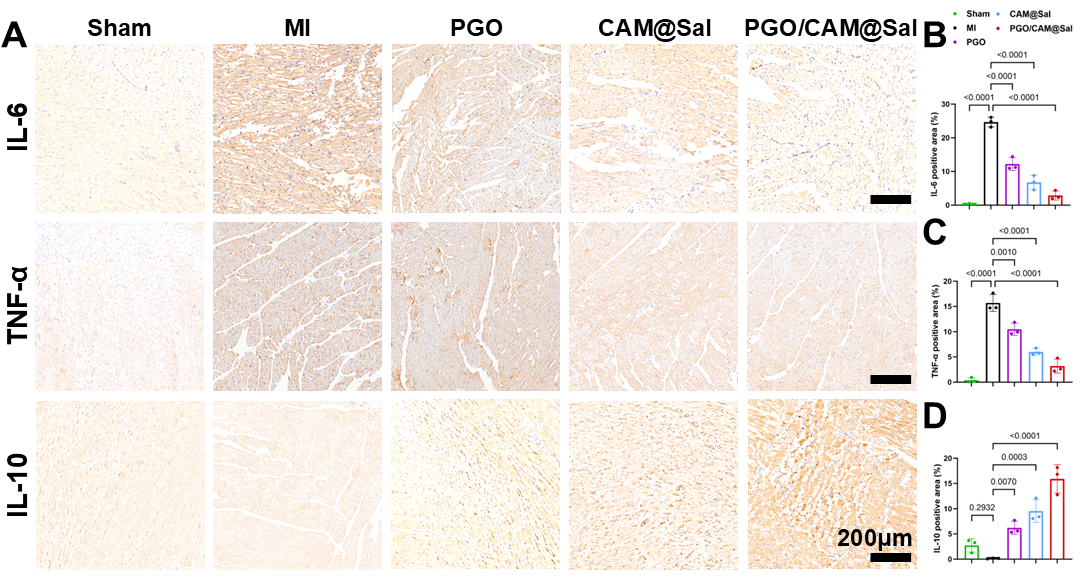


**Figure S16.** Immunohistochemical staining of IL-6, TNF-𝛼, and IL-10 in rat hearts (A） and quantitative analysis (B-D) using ImageJ (n = 3).


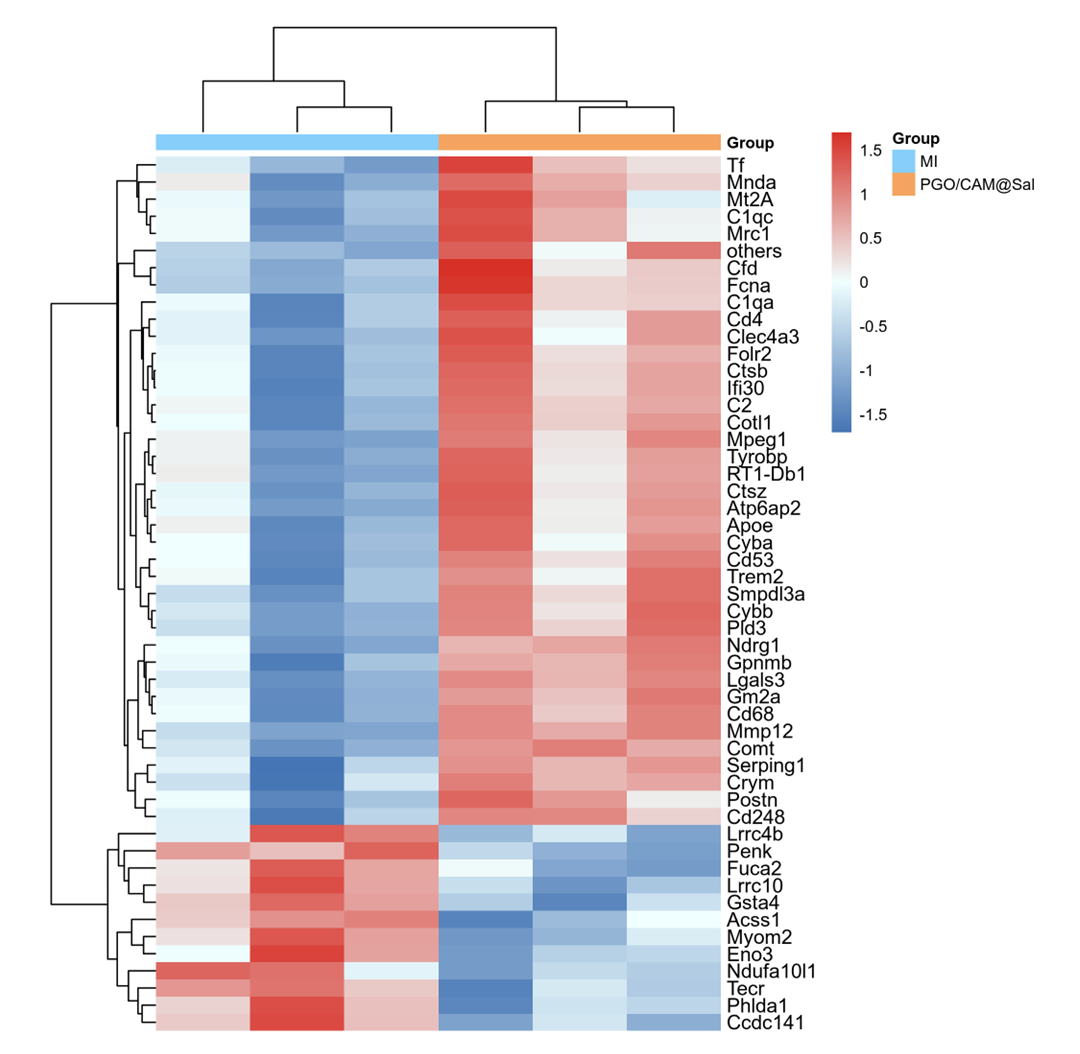


**Figure S17.** Heatmap of the top 50 differentially expressed genes (DEGs) between the MI and PGO/CAM@Sal groups (*p* < 0.05 and |fold change| > 2).


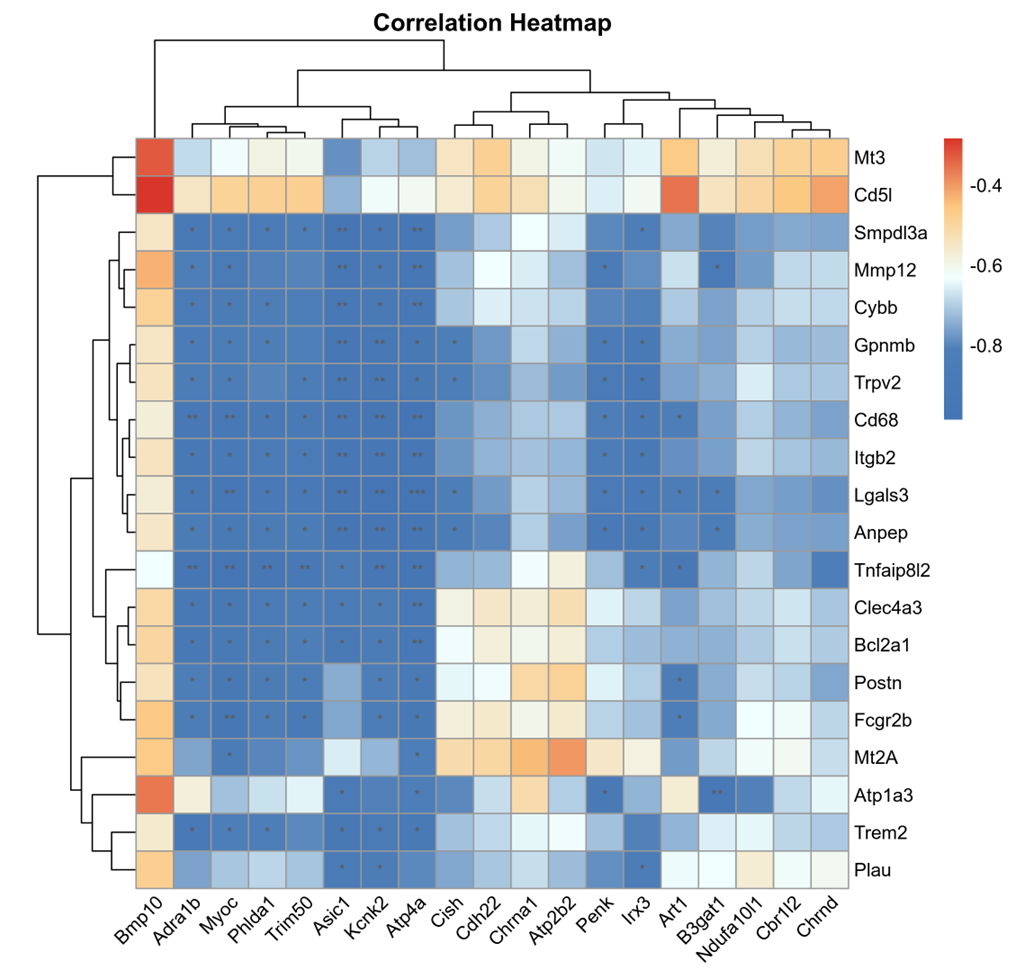


**Figure S18.** Co-expression correlation heatmap of upregulated genes in the MI and PGO/CAM@Sal groups (**p*＜0.05, ***p*＜0.01, ****p*＜0.001).


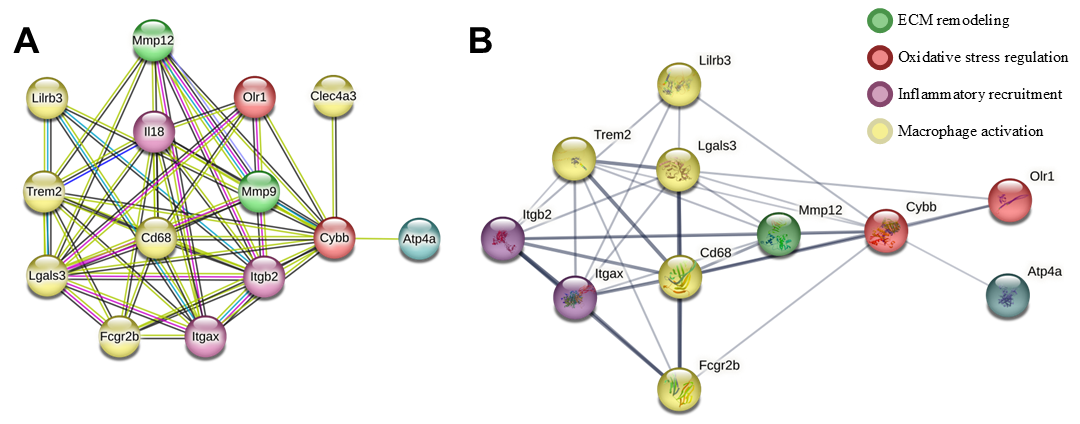


**Figure S19.** PPI network centered on Cybb/Atp4a (A）and MMP12/Cybb (B).
